# Whole-exome analysis in Tunisian Imazighen and Arabs shows the impact of demography in functional variation

**DOI:** 10.1038/s41598-021-00576-0

**Published:** 2021-10-26

**Authors:** Marcel Lucas-Sánchez, Neus Font-Porterias, Francesc Calafell, Karima Fadhlaoui-Zid, David Comas

**Affiliations:** 1 Departament de Ciències Experimentals i de la Salut, Institut de Biologia Evolutiva (CSIC-Universitat Pompeu Fabra), Universitat Pompeu Fabra, Barcelona, Spain; 2 Laboratory of Genetics, Immunology, and Human Pathologies, Faculty of Science of Tunis, University of Tunis El Manar, Tunis, Tunisia; 3 College of Science, Department of Biology, Taibah University, Al Madinah Al Monawarah, Saudi Arabia

**Keywords:** Genetic variation, Biological anthropology

## Abstract

Human populations are genetically affected by their demographic history, which shapes the distribution of their functional genomic variation. However, the genetic impact of recent demography is debated. This issue has been studied in different populations, but never in North Africans, despite their relevant cultural and demographic diversity. In this study we address the question by analyzing new whole-exome sequences from two culturally different Tunisian populations, an isolated Amazigh population and a close non-isolated Arab-speaking population, focusing on the distribution of functional variation. Both populations present clear differences in their variant frequency distribution, in general and for putatively damaging variation. This suggests a relevant effect in the Amazigh population of genetic isolation, drift, and inbreeding, pointing to relaxed purifying selection. We also discover the enrichment in Imazighen of variation associated to specific diseases or phenotypic traits, but the scarce genetic and biomedical data in the region limits further interpretation. Our results show the genomic impact of recent demography and reveal a clear genetic differentiation probably related to culture. These findings highlight the importance of considering cultural and demographic heterogeneity within North Africa when defining population groups, and the need for more data to improve knowledge on the region’s health and disease landscape.

## Introduction

North Africa has an advantageous location, joining the Mediterranean Sea, the Sahara Desert, and the Middle East. Because of this strategic position, North Africa has been the destination or pathway of many demographic movements involving surrounding regions, shaping the genomes of its inhabitants as a complex amalgam of different ancestral components^1–4^. Since the first known human presence in North Africa, dated to around 300,000 years old^5,6^, different pre-Neolithic cultures succeed each other in the archaeological record until the arrival of the Neolithic from the Middle East^7–13^, although genetic continuity in the region has been demonstrated from at least the Paleolithic^3,14,15^. In historical times, several Mediterranean populations sequentially arrived in North Africa (Phoenicians, Greeks, Romans, Vandals and Byzantines)^7,16,17^, although their genetic and cultural impact in the region was limited^2,3^. In the seventh century, the Arabs conquered North Africa from the Middle East and, in contrast, had a strong influence in North African peoples that still lasts today, despite later arrivals of the Ottoman Empire and European colonial powers^7,16,17^. Present day North Africa exhibits thus a complex cultural landscape, with a rich diversity of languages, religions, and cultural practices. Its inhabitants have been traditionally divided in two main groups, Arabs and Berbers, a misnomer that traces back to Greco-Roman times (from the Latin word *barbarus,* babbling foreigner) to designate the original inhabitants of the region^18,19^, who identify themselves as Amazigh (sing.)/Imazighen (pl.) (free people)^20^. Imazighen are considered the descendants of Paleolithic North Africans^7,21,22^, and although pre-Arabic incursions did not have a large demographic impact on them, the arrival of the Arabs and mainly the massive Bedouin immigration in the eleventh century had a strong cultural importance on Amazigh populations in language, religion, and customs^7,16,17^. Most North Africans incorporated the new culture, admixed with the newcomers, and began to identify themselves as Arabs, but others escaped this influence, receding to remote and isolated villages, where they maintained their original culture, language (Tamazight), and Amazigh identity^20,23–26^.

Thus, several Amazigh groups are believed to have been affected by severe bottlenecks followed by geographic and genetic isolation since the Arab expansion, leading to small effective population sizes^23,24,27,28^. In contrast, other Amazigh groups exhibit similar genetic diversity as non-Amazigh populations^2,3^, challenging the notion of Imazighen as a single homogeneous group. In this sense, although two gene-expression studies have been performed to clarify differences among Amazigh populations with different lifestyles in a region of Morocco^26,29^, to our knowledge no study has yet assessed the impact of the mentioned demographic events in exome-wide functional differences, characterizing the distribution of variants and its possible biomedical effects in North African populations, until now.

Small populations, compared to larger ones, are predicted to be more affected by genetic drift (i.e. random fluctuations in allele frequencies), have lower genetic diversity and, as a consequence, higher genetic load, defined as the reduction in mean fitness in a population relative to the theoretical maximum fitness^30–34^. Specifically, most genetic load studies measure the mutational load, which is the genetic load caused by the accumulation of deleterious mutations relative to a mutation-free population. Also, purifying selection is predicted to be less efficient in removing deleterious alleles in small populations^35^. Different populations affected by similar events have been studied^36–44^, with contrasting conclusions. On one hand, some studies suggest that a decline in effective population size leads to a decrease in genetic variation, thus reducing the substrate for purifying selection to act upon and lowering its efficacy while increasing the effect of genetic drift. This would cause the accumulation of slightly deleterious alleles and homozygous derived genotypes in a higher proportion than in non-affected populations^36–38,43,44^. On the other hand, other studies showed that Europeans and sub-Saharan Africans carry the same amount of derived alleles, despite the severe out-of-Africa bottleneck experienced by Europeans, concluding that genetic load is not affected by recent size changes^39,40^. Part of the discrepancy lies in how the effect of selection was measured, as there is no universally accepted metric of genetic load or selection efficacy in human populations^43^. However, many studies have used summary statistics to approximate the mutational load under different allele dominance models: the average number of derived alleles per individual (N_alleles_) to calculate the load assuming an additive model (i.e. all alleles have an additive effect), and the average number of homozygous derived genotypes per individual (N_hom_) when assuming a recessive model (i.e., all alleles have a recessive effect)^36–38^. It should be noted that, as mentioned, the arguments about the lack of effect of recent demography on genetic load are based mostly on comparisons between populations of European and sub-Saharan African ancestry^39–44^. Studies about populations with a much deeper or recent effect of such demographic events^36,45,46^ agree in the accumulation of deleterious alleles and homozygous genotypes, the increased recessive load, and a shift in variant distribution, with fewer rare and more common variants. They also point to relaxed purifying selection in such populations.

In the present study, we carried out the first analysis on the distribution of variants and its functional consequences in populations living in isolated locations in the complex demographic landscape of North Africa. We studied the Tunisian Amazigh population from two geographically isolated villages in the Tataouine governorate in southern Tunisia, comparing this group to a sample of non-Amazigh individuals from the city of Tunis. We studied high coverage whole-exome sequences from 18 Amazigh and 46 non-Amazigh individuals to test their population structure differences arising from their different demographic histories, and assess their genetic consequences focusing on the frequency distribution of functional variants and their biomedical implications. Our results show the sharp differences in variant distribution and their potential effects on health and disease between the two neighbor populations as a consequence of a distinct demographic background and highlight the significant need for more biomedical and functional studies in North Africa.

## Results

### Population structure and demography from exome variants

**Figure 1:**
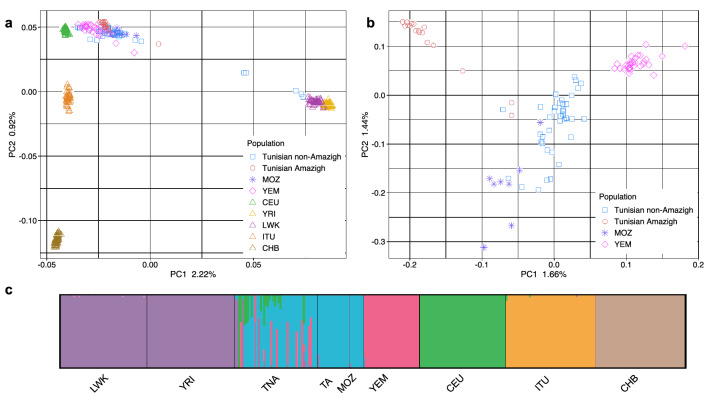
Principal component analysis and ADMIXTURE analysis for *K* = 6. (**a**) Principal component analysis of exome variants in Tunisian samples together with a panel of surrounding and other worldwide populations. (**b**) Principal component analysis of Tunisian samples together with Mozabite and Yemeni samples, excluding nine outlier individuals in A. (**c**) ADMIXTURE analysis (K = 6) of North African samples together with a panel of surrounding and other worldwide populations. Population abbreviations stand for Tunisian Amazigh (TA), Tunisian non-Amazigh (TNA), Mozabite (MOZ), Yemeni (YEM), Yoruba (YRI), Luhya (LWK), Utah residents (CEPH) with Northern and Western European ancestry (CEU), Han Chinese in Beijing (CHB) and Indian Telugu in the UK (ITU).

We produced 75 whole-exome sequences from Amazigh and non-Amazigh Tunisians, 64 of which passed the relatedness filter, yielding a total of 319,297 SNPs (see “Materials and methods”). The population structure of the present Tunisian exomes was assessed through principal component (PC) and ADMIXTURE analyses including other populations as references (Fig. 1). The first two principal components of the exome variants (Fig. 1a) show a Europe-sub-Saharan Africa cline in PC1, with North Africans in the middle but closer to the European and Middle Eastern groups. The ADMIXTURE analysis performed (Fig. 1c, Supplementary Figs. S1 and S2 online) shows Tunisian non-Imazighen to have a mixed pattern with a main North African-related component and some traces related to our sub-Saharan, Middle Eastern, and European proxies, while Tunisian Imazighen show a more homogeneous ancestry pattern similar to that shown by Mozabites, an Algerian Amazigh group. These results from the exome variants agree with the previously known genomic structure of the populations in our dataset^1–3^, and show both Tunisian groups clustering separately from other populations but with some internal differences between Imazighen and non-Imazighen (Fig. 1b). Tunisian Imazighen appear separated from the other two North African populations, which instead show some overlap (Fig. 1b). Recent sub-Saharan gene flow^1,2^ might be the cause of the outlier position of some Tunisian non-Amazigh individuals (Fig. 1). Similar results were obtained when these were removed from subsequent analyses. Pairwise Fst distances between populations match the PCA and Admixture results (Supplementary Fig. S3 online).

**Figure 2:**
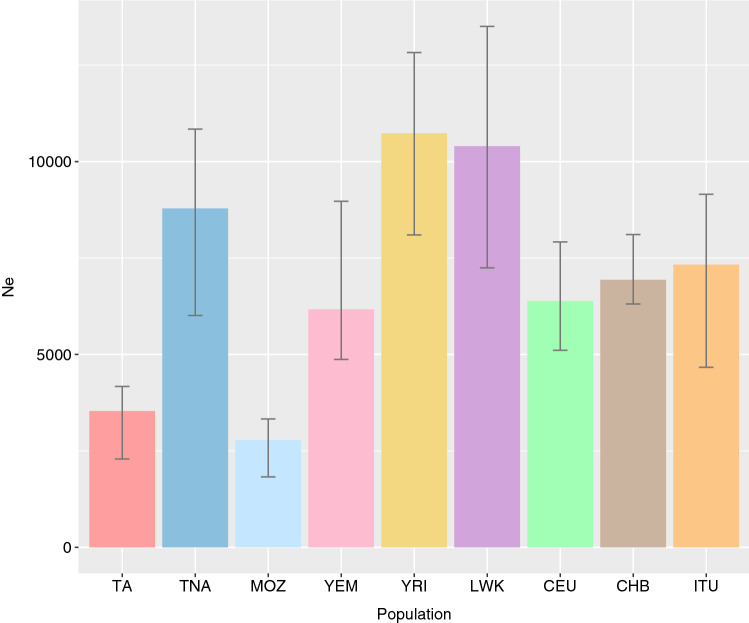
Long-term effective population size. Long-term N_e_. Error bars indicate the 5th and 95th percentile of the distribution using each chromosome as replicates. Population names abbreviated as in Fig. 1.

In order to assess the effective population size (N_e_) of the present sample set, we explored N_e_ history based on patterns of linkage disequilibrium (LD) with the whole-exome data^47^. The analysis of long-term N_e_ (the harmonic mean of N_e_ along the past generations explored) confirms that both Tunisian Amazigh and Mozabite populations have significantly lower long-term N_e_ than the rest of populations (Fig. 2). Amazigh and non-Amazigh Tunisian groups show notable differences in their estimated N_e_ values. Along the same lines, a large effect of genetic drift in the Tunisian Amazigh population was revealed by the TreeMix analysis (Supplementary Fig. S4 online).

### Runs of homozygosity

A clear revealing element of past or present demographic history involving small population sizes is the amount and length of runs of homozygosity (ROHs). Long runs of homozygosity point to recent inbreeding and consanguinity, being created when identical haplotypes are inherited from each parent, i.e., when related individuals have descendants. Because of their low effective population size, this is usual in small and genetically homogeneous populations, which are also highly affected by genetic drift. On the other hand, shorter runs of homozygosity reflect a history of past rather than recent small population sizes^48^.

**Figure 3:**
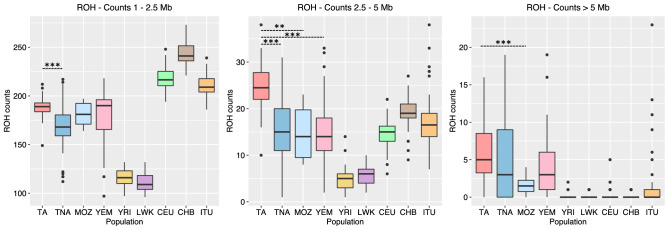
Per-individual counts of runs of homozygosity in different length categories. Boxplots indicate the distribution of the per-individual number of runs of homozygosity in different populations. Points indicate outlier individuals. Dashed lines indicate a statistically significant t-test between Tunisian Imazighen and other North African and Middle Eastern populations. Statistical significance is shown in the following way: *p < 0.05, **p < 0.01, ***p < 0.001. Population names abbreviated as in Fig. 1.

After classifying ROHs by length categories, Tunisian Imazighen exhibit significantly higher per-individual number of ROHs than Tunisian non-Imazighen (Fig. 3), and they present the highest values for ROHs 2.5–5 Mb long in the whole dataset (significant p-values for all population pairs). Tunisian Imazighen also present the highest mean for per-individual ROHs longer than 5 Mb, although this value is not significant when compared to Tunisian non-Imazighen and Yemenis due to the lower number of ROHs in this category. Similar results were found when analyzing the per-individual total and average ROH length (Supplementary Figs. S5 and S6 online).

ROH analyses show that Tunisian Imazighen present long continuous tracts of homozygous sites, notably differentiated from Tunisian non-Imazighen, probably reflecting recent inbreeding as a result of isolation, and concordant with N_e_ results (Fig. 2).

### Genetic diversity and mutational load

**Figure 4:**
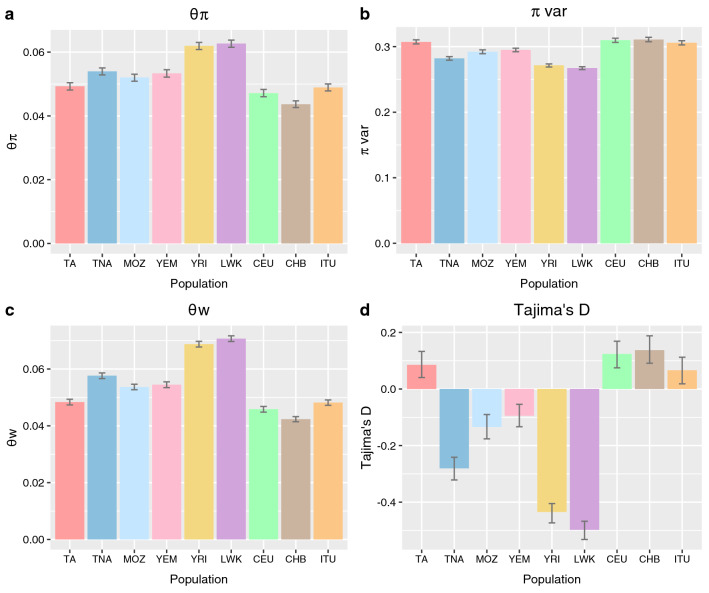
Genetic diversity indexes. (**a**) Pairwise nucleotide diversity θπ. (**b**) Nucleotide diversity for the variable sites πvar. (**c**) Watterson’s estimator θw. (**d**) Tajima’s D. Error bars represent the 0.025 and 0.975 quantiles obtained by bootstrapping by site 1000 times, dividing the exome data into 1000 blocks and performing bootstrap resampling of blocks 1000 times. Population names abbreviated as in Fig. 1.

Following N_e_ results (Fig. 2), as small populations are predicted to have lower genetic diversity^30,32–34^, we calculated four different diversity indexes for all populations in our dataset: pairwise nucleotide diversity (θ_π_); nucleotide diversity for variable sites (π_var_) , which is predicted to follow an opposite pattern from θ_π_^36^_;_ Watterson’s estimator (θ_w_); and Tajima’s D (Fig. 4). All four indexes confirmed that Tunisian Imazighen are significatively (*p* < 0.001) less genetically diverse than the rest of North African and Middle Eastern populations analyzed, as we would expect considering their N_e_ and demographic history. Consistent with these findings, Tunisian Imazighen have a lower average number of segregating sites per individual than the rest of North Africans and Yemenis, and less population-specific segregating sites per individual than the rest of populations analyzed (Supplementary Table S1 online), confirming their reduced genetic diversity.

**Figure 5:**
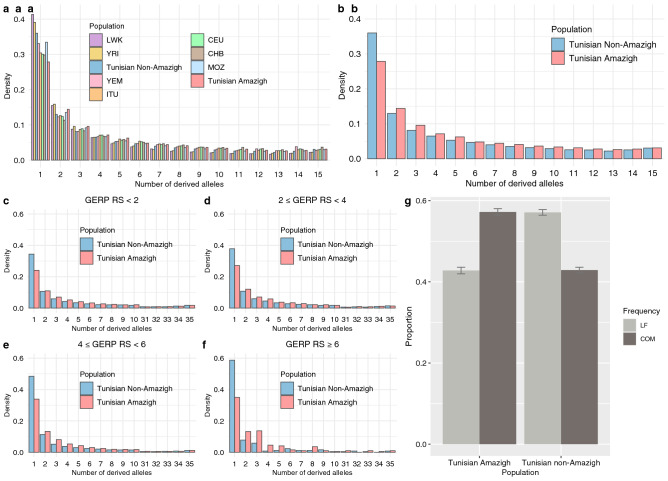
Site frequency spectra of derived alleles and derived allele proportions. Each population was subsampled to 18 individuals, and fixed sites were excluded. For Tunisian Imazighen, the 18 individuals selected were all of the available in our dataset. For Tunisian non-Imazighen, 18 randomly selected individuals were used. (**a**) General site frequency spectrum derived alleles for Tunisian populations, together with a panel of surrounding and other worldwide populations. Population names abbreviated as in Fig. 1. (**b**) General site frequency spectrum of derived alleles for Tunisian populations. (**c**–**f**) Site frequency spectra of derived alleles in different GERP RS score categories. Plot title indicates the range of GERP RS scores of the variants included. (**g**) Proportion of derive deleterious variants classified by frequency-based categories. Variants included are those with GERP RS scores higher than 2. The frequency-based categories are Low-Frequency (LOW), including singletons and doubletons, and Common (COM), including frequencies higher than tripletons. Error bars represent the 95% confidence intervals.

To further evaluate the genetic consequences of the different demographic histories of Tunisian Amazigh and non-Amazigh populations, we explored the site frequency spectrum (SFS) of functional variation, which is significantly affected by demographic history^36,49,50^. Tunisian Imazighen exhibit a flatter SFS than all other populations, with a lower frequency of rare alleles and an increase in the common frequency categories (Fig. 5a). This difference is remarkable when comparing both Tunisian groups (Fig. 5b). Such a decrease in the proportion of low-frequency variants can be a result of a strong bottleneck or inbreeding, or, most likely, a combination of both events followed by drift.

In order to explore these patterns specifically in putatively deleterious variation, we compared the unfolded SFS between populations for different categories of deleteriousness using GERP RS scores^51,52^. We used the same categorization for deleteriousness of the variants proposed in^38,53^: “neutral” (GERP < 2), “moderate” (2 ≤ GERP < 4), “large” (4 ≤ GERP < 6), and “extreme” (GERP ≥ 6). The pattern observed in the general SFS is similar when dividing sites according to their GERP RS scores, with Tunisian Imazighen having fewer variants in the singleton category but more in the common frequency bins (Fig. 5c–f, Supplementary Fig. S7 online). Moreover, this difference seems to increase as the predicted deleteriousness of the GERP RS category increases. This trend between Amazigh and non-Amazigh Tunisians (Fig. 5c–f) was confirmed with bootstrap analyses for the first ten categories of the SFS (see “Materials and methods”), corroborating that the higher the predicted deleterious effect, the higher the differences between both Tunisian populations (Supplementary Table S2 online). This can be indicative of a decrease in the efficacy of purifying selection in purging the more deleterious mutations in the Amazigh population, which then reach higher frequencies, as a result of higher genetic drift.

When the number of non-neutral variants (i.e., GERP ≥ 2) were pooled together and divided into low-frequency variants (singletons and doubletons) and common variants (Fig. 5g), we observed that Tunisian Imazighen present a higher proportion of their predicted deleterious variation in common frequency than in singletons or doubletons, remarkably different than Tunisian non-Imazighen and concordant with the previous SFS results. Additional methods to assess the deleteriousness of the variants (i.e., CADD and Polyphen scores) were used giving similar results as the GERP RS scores (Supplementary Figs. S8 and S9 online), although the limited number of variants predicted as deleterious or damaging by Polyphen (42,505) compared to GERP (114,537) or CADD (141,656) resulted in an enrichment of rare variants in the PolyPhen analysis. Thus, Tunisian Imazighen present a larger number of common deleterious variants than Tunisian non-Imazighen in all three deleteriousness classifications.

**Figure 6:**
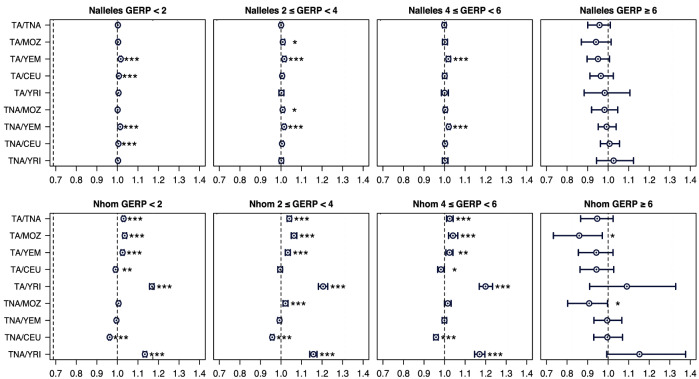
Comparison of the per-individual number of derived alleles (N_alleles_) and homozygous derived genotypes (N_hom_) across populations for variants in different GERP RS score categories*.* Pairwise population ratios of the mean per-individual number of derived alleles and homozygous derived genotypes. Plot title indicates the range of GERP RS scores of the variants included. Error bars represent the 0.025 and 0.975 quantiles obtained by bootstrapping by site 1000 times, dividing the exome data into 1000 blocks and performing bootstrap resampling of blocks 1000 times. Statistical significance is shown in the following way: *p < 0.05, **p < 0.01, ***p < 0.001. To account for multiple testing errors, significance threshold was set to *p* < 0.001. Population names abbreviated as in Fig. 1.

To have a richer view of the burden and distribution of deleterious variation in the studied populations, we estimated the mutational load across them (Fig. 6). Under the assumption of an additive model (N_alleles_), we observed a significant but very slight increase in the mutational load of both Tunisian populations in comparison with Yemenis (*p* < 0.001 for the first three GERP categories) and, in the “neutral” category (GERP < 2), with Europeans (*p* < 0.001).

When assuming a recessive model (N_hom_), mutational load is significantly higher in Tunisian Imazighen than in Tunisian non-Imazighen, Mozabites, Yemenis and Yoruba for the “neutral” (GERP < 2) and “moderate” (2 ≤ GERP < 4) GERP RS score categories (*p* < 0.001), and this is maintained in all comparisons with the exception of Yemenis in the “large” GERP RS score category (4 ≤ GERP < 6). The more deleterious GERP RS score category (GERP ≥ 6) contains much fewer variants than the others (2172 compared to 57,236 in 4 ≤ GERP < 6 and 55,129 in 2 ≤ GERP < 4) as it assumes an extremely deleterious effect for variants, which means that even in small populations these variants are more likely to be removed by purging selection. This is probably causing the large confidence intervals in this category for both models.

The load calculation was also performed applying a second filter to variants by selecting only the ones labeled as missense in the Ensembl Variant Effect Predictor^54^ for the ones with GERP RS score ≥ 2, and sites labeled as synonymous for variants with GERP RS score < 2, as performed in^37^ (Supplementary Fig. S10 online). The load trend was the same as shown above, but statistical significance was slightly reduced in some pair comparisons as a result of the reduced number of variants when applying both filters (114,536 variants with GERP ≥ 2 compared to 68,839 variants with GERP ≥ 2 and a missense label, and 192,406 variants with GERP < 2 compared to 58,659 variants with GERP < 2 and a synonymous label), as only 184,060 of the 306,942 variants in our dataset are labelled as synonymous or missense in the Ensembl Variant Effect Predictor^54^.

When mutational load was assessed through additional deleteriousness approaches (CADD and PolyPhen scores) similar results were found (Supplementary Fig. S11 online). As an additional analysis, we also calculated another statistic in this same line, the GERP score load, which showed similar results as the ones here presented (Supplementary Fig. S12 online).

Related to these results, our ROH analyses show that across most populations and particularly in Tunisian Imazighen, the ratio of deleterious to synonymous homozygous sites is higher inside ROHs than outside (Supplementary Figs. S13, S14, S15 online), especially for sites with 4 ≤ GERP RS scores < 6 (*p* < 0.0001). These findings suggest that a higher number of ROHs, as seen in Tunisian Imazighen (Fig. 3), results in an accumulation of homozygous damaging sites, with its potential biomedical effects, and are in concordance with the previous evidence of enrichment for homozygous deleterious variants in ROH regions^48,55,56^ and with our findings regarding genetic load (Fig. 6). The presence of more and significantly longer runs of homozygosity in Tunisian Imazighen can have important biomedical implications because long ROHs are known to be involved in predisposition to both rare and common diseases^48^.

### Enrichment analysis and biomedical significance

Aiming to gain further insight into the implications for health and disease of the observed differences between Amazigh and non-Amazigh populations in Tunisia, we conducted a set of enrichment analyses based on the differential allele frequencies for possibly deleterious variants. Fst values between Imazighen and non-Imazighen were calculated for each site in our dataset, and those labeled as missense and having GERP RS scores ≥ 2 or predicted as damaging in PolyPhen were selected (details in “Materials and methods”). For these analyses, we focused on those traits that are enriched in Imazighen, since our previous results reveal a higher accumulation of homozygous sites (Fig. 3) and putatively deleterious variation (Figs. 5, 6) than in non-Imazighen, which could underlie unique biomedical consequences. Only variants with higher derived allele frequencies in the Amazigh population were kept. Because these variants are not present in the main public databases (with extremely few exceptions), which are built using mainly European data^57,58^, we translated them to their corresponding genes as an approximation and used these gene sets in four different databases suitable for enrichment analysis (Supplementary Table S3 online).

The enrichment analyses using the Online Mendelian Inheritance in Man (OMIM) catalog^59^ identified several gene associations with relevant health conditions (Supplementary Tables S3 and S4 online). For hypertension (MIM: 145500), one variant with significantly higher frequency in the Tunisian Amazigh population reported as risk factor in the OMIM database (Supplementary Table S5 online) was found. One additional reported risk variant was also found for obesity (MIM: 601655), although the enrichment of this disease only matched one of the criteria for the analysis (*p* < 0.05). For most traits (including hypertension), we found missense deleterious variants with significantly higher frequencies in Tunisian Imazighen whose effects have not yet been studied, although they are found in genes related to the corresponding condition (Supplementary Table S5 online).

Genes reported in the GWAS catalog^60^ and with variants enriched in Imazighen included many genes associated with traits related to bone and anatomy (height, body mass index, adolescent idiopathic scoliosis, bone mineral density, arthritis, etc.), some to respiratory function (asthma, lung function), blood related traits (blood protein levels, red and white blood cell count) and other conditions such as metabolite levels, colorectal cancer, inflammatory bowel disease or type 2 diabetes (Supplementary Tables S3 and S6 online). These traits were significantly enriched even when correcting by the trait frequencies in the GWAS catalog.

The ConsensusPathDB (CPDB) program^61^ was used to explore the enrichment of biological pathways rather than diseases, and the main results can be roughly grouped in four sets: the olfactory function (including related G-protein signaling), extracellular matrix (collagen related pathways), fatty acid metabolism, and rRNA processing, with a single IL-12-related pathway not linked with the previous groups (Supplementary Tables S3 and S7 online). Additional enriched pathways include pathways related to inositol metabolism or endocytosis, among others.

Finally, the enriched pathways resulting from the Gene Ontology (GO) Resource^62,63^ analysis included, among others, cholesterol-related traits, coherent with the OMIM results, although only a number of pathways that can be grouped under the umbrella of olfactory function and signaling meet our top significance criterion (FDR < 0.05) (Supplementary Tables S3 and S8 online).

To look for additional specific examples of the results obtained with the enrichment analyses we checked the genes in the peaks of the Fst distribution, as defined in “Materials and methods”, which include groups of highly differentiated variants, most times in the same gene. In these peaks, we found several genes that have been related to bone conditions both in the GWAS catalog and in the general literature. In three of the four peaks reported in Supplementary Table S9 online, SNPs were separated less than 1 kb from each other, suggesting a possible strong association between these highly differentiated sites. This strong association was confirmed by the high r^2^ found between SNPs in these peaks (Supplementary Table S10 online). We also checked the possible biomedical associations of highly differentiated variants between both Tunisian populations with very high GERP RS scores and report them in Supplementary Table S11 online. Several bone conditions were also found, as well as other potentially interesting traits such as asthma, intraocular pressure, or the Aicardi-Goutières syndrome, among others.

## Discussion

The genetic functional consequences of demographic processes have been described and debated in several human population groups^24,36,37,45–47,56^. Within this framework, we have conducted the first study on the impact of demography in the distribution of functional variation in North African populations, being also one of the few works focused on biomedical function in North Africa to take into account the cultural differences of the studied populations (in our case, Amazigh and non-Amazigh), and the first to use our approach in the region. We provide and characterize new high depth whole-exome sequences of North African Amazigh and Arab populations, making this the first whole-exome study with North Africa as the main region of study.

The main aim of our study was to carry out a genomic characterization of a rural and isolated Amazigh population in the complex demographic context of North Africa (Tunisian Imazighen from two villages in the Tataouine governorate), focusing on variant distribution, and comparing it with a non-isolated non-Amazigh sample of individuals from the city of Tunis. These two populations have experienced different demographic histories, with the main divergence being relatively recent, in the context of the Arab expansion of North Africa (seventh–eleventh centuries)^4,7,16,17,25^. Hence, we show here the impact of recent demography on human genomes from a functional point of view. Our results confirm that these different population histories have resulted in relevant genetic differences which in turn might lead to distinct biomedical implications. The Amazigh group shows signs of genetic isolation, possibly after a relatively recent bottleneck, and followed by strong genetic drift and inbreeding. We also find evidence of admixture in Tunisian non-Imazighen in more relevant proportions than in the Imazighen group, which might also have an effect in the observed results. The estimations of effective population size and the diversity indexes confirm the particular demographic situation of the population and its sharp difference with the non-Amazigh individuals. As a consequence, Tunisian Imazighen carry significantly more and longer runs of homozygosity than their non-Amazigh neighbors, and a larger fraction of their variants are found at high frequencies, exhibiting thus depletion of rare variants in comparison with Tunisian non-Imazighen. Moreover, this pattern is maintained when assessing only variants with predicted deleterious consequences, which indicates that a higher proportion of variants with a potential damaging biomedical effect can reach a considerably higher frequency in the Amazigh population. Our approximation to mutational load is also in the line of these results, as Tunisian Imazighen exhibit a significantly higher load under a recessive model of dominance, i.e., when the summary statistic used to calculate the mutational load is the per-individual number of homozygous derived genotypes. It is important to note that genetic load cannot be directly calculated from whole-exome data^41^ and that the summary statistics approach cannot be read as a perfect reflection of real genetic load, but under this widely used approximation we observe results coherent with those arising from the runs of homozygosity and SFS analyses.

These findings lead to the debate of the efficiency of purifying selection in isolated or bottlenecked populations such as the Tunisian Imazighen. Whether selection is affected by changes in population size has been consistently studied and debated over the years^36,38–40,42,44–46,64^. Here we approached the question with two main lines of analyses: (i) SFS and distribution of deleterious variation, and (ii) an approximation to mutational load. The SFS is affected by recent demographic history, and its comparison across populations can reveal differences in the effect and strength of genetic drift and natural selection^36,49,50^. As mentioned above, we observe in the SFS that Tunisian Imazighen exhibit a higher proportion of putatively deleterious alleles at high frequencies than non-Imazighen and other non-isolated populations. Concordant with these results and in agreement with previous evidence^36,37^, the approximation to mutational load under a recessive model (i.e. the ratio of homozygous derived genotypes) also shows that the Amazigh population exhibit a higher accumulation of homozygous derived genotypes across different deleterious categories. Such differences suggest that, at least under a recessive model, purifying selection may have had a lower impact in removing possibly damaging variation, allowing for this to reach higher frequencies in the isolated population. This is probably a result of Tunisian Imazighen having a much larger effect of genetic drift, an evolutionary force with opposite effect to purifying selection^34,65–67^. The increased effect of genetic drift leads to lower diversity, thus reducing the substrate for purifying selection to act and decreasing its efficiency^44,68^. Most studies framed in the debate on the relaxation of purifying selection after a bottleneck or isolation have focused on the out-of-Africa bottleneck and the comparison between European and sub-Saharan African populations^39–44^. In these cases, the time passed since the event and the growth of European populations may impinge on the actual effect of the bottleneck. The isolation in Tunisian Imazighen, in contrast, is much more recent and lacks the expansion of European populations which makes it difficult to compare with the out-of-Africa situation. Our results are concordant with other studies in populations with comparable historical isolation or extreme bottlenecks like the Inuit^36^, the Finns^45^, or the French-Canadians^46^, suggesting a possible relaxation of purifying selection in populations affected by such events.

Population isolates have been widely studied for their potential in mapping and identifying disease- and trait-related genes and variants^36,69–74^. This is because these populations have high genetic and environmental homogeneity, and variants involved in specific diseases and quantitative traits are sometimes present at higher frequencies than in other populations, increasing the power for association studies. Because of the observed variant distribution of Tunisian Imazighen and the high number of potentially damaging variants at high frequencies, we believe that they constitute a suitable candidate for that type of analysis in the complex context of North Africa. This would provide deeper knowledge on the disease risk and disease prevalence pattern in North Africa, which would have direct beneficial consequences on the health of its inhabitants, while also having the potential to discover new variant associations with impact outside of North African borders, given the extensive gene flow from North Africa to its surroundings^75,76^.

To assess the possible specific biomedical implications of the observed particular variant distribution in Tunisian Imazighen, we have conducted enrichment analyses examining different sets of variants with the highest frequency difference between both Tunisian populations, specifically those with higher frequencies in Imazighen. Our results revealed the enrichment of different traits in Tunisian Imazighen, with a higher presence of those related to bones and anatomy, respiratory and blood functions, and the olfactory function. This last case is driven by the presence of olfactory receptors and related genes, which vary substantially among populations or even individuals as a result of genetic drift, selective pressures and, more importantly, pseudogenization processes^77–80^. This makes us cautious about the interpretation of this specific result. Taken together, our results indicate that the genetic differences between Tunisian Amazigh and Tunisian non-Amazigh populations could also be the underlying cause of differences in their health and disease profile, i.e., that the particular genetic structure and variant distribution in Tunisian Imazighen could have biomedical consequences different from those of the urban non-Imazighen population. Other non-genetic factors, such as social factors, could also be involved in biomedical differences between these populations, but further data would be needed to fully cover this subject.

The main barrier that challenges genetic studies in North Africa is the scarce genetic data available. Despite our interesting findings on conditions, traits, and biological pathways that could present relevant differences between Imazighen and non-Imazighen in Tunisia, there is an urgent need for more phenotypic and disease prevalence data in North African populations. The question remains on whether there is a higher prevalence, risk or drug response for the reported traits and diseases in Tunisian Imazighen as, because of the scarce data, we cannot link these results to previously published biomedical and phenotypic reports in North Africa, which often describe a general population with no attention to cultural differences. In this regard, we have found in our enrichment analyses several missense or even some stop-gain variants whose consequences have not been studied, and do not appear in the corresponding databases, which are clearly biased towards the more studied European-descent populations^57,58^. More (and more specific) biomedical studies are needed to discern this matter. We believe our results can serve as a starting point for future studies targeting this question that will help to continue describing the biomedical landscape of North Africa, including the differences between its heterogeneous population groups.

Although exomes represent only a part of the genome, our population structure results are in agreement with those obtained with genome-wide data^1–3^, and reinforce the idea of a recent and possibly culture-driven differentiation of the two groups, rather than a difference in their historical origin. Thus, we see here how a socio-cultural event (the Arab expansion) has led to significant differences in the genomic configuration of two populations in a relatively small territory. We present a new perspective to the culture-genetics correlation debate in North African populations, compatible with previous evidence and more biomedically- or functionally-focused than the previous admixture-based studies. As other authors pointed^2,3^, not all Tamazight-speaking groups are isolated, and Imazighen are in fact a diverse group of populations with a common origin but different population histories. Nonetheless, some of them are more isolated, probably because of a different impact of Arabization. Our results show how a North African population that maintained an Amazigh identity and way of life has undergone distinct demographic processes not experienced by another population that identifies as Arab, and that these events have influenced their genetic architecture. As revealed in previous studies, genetics and cultural identity do not always go side by side in North Africa, but in the two populations studied in our work we see a clear genetic differentiation with possible socio-cultural causes. For this and other groups, isolation is a distinct and shared trait that differentiates them from Arabs, Arabized Imazighen, and urban Imazighen. Isolation and genetic drift are main drivers of population differentiation^32,67^, creating a continuous range of genetic diversity, and they have been of crucial importance in shaping the demographic landscape observed nowadays in North Africa. Isolation in North Africa should then be considered with the same importance as differential admixture and taken into account when defining population groups for biomedical studies. In the light of our results, we believe future studies should differentiate between these different groups of populations in North Africa. We believe more data on new Amazigh and Arab populations is needed, and more North African groups (Amazigh, Arab, isolated, and non-isolated) should be studied following an approach similar to the one here presented, to go deeper in this question and to have a richer knowledge of the cultural and genetic diversity of North Africa, and the relations between them.

## Materials and methods

### Ethics declaration

Written informed consent was obtained from all the volunteers and the present project has the corresponding IRB approval (CEIC-Parc de Salut Mar 2019/8900/I). All the methods were carried out in accordance with relevant guidelines and regulations.

### Individuals selection and sample collection

North African samples from two Tunisian populations were sequenced in the present study: Amazigh individuals from two villages in the Tataouine governorate in southern Tunisia (n = 20) that were pooled together because of their geographical closeness (~ 20 km apart), and non-Amazigh individuals from the city of Tunis (n = 55). Participants were healthy volunteers with appropriate informed consent. Amazigh individuals were Chela-speakers, a language in the Tamazight family, while non-Imazighen were Arab-speakers. Blood samples were collected for non-related individuals in these populations and whole-exome sequencing was performed with the Agilent SureSelect Human All Exon V6 capture kit.

### Data processing and quality controls

Raw sequencing data was stored in FASTQ files, which were then trimmed to remove adaptor sequences using Trimmomatic^81^. Read quality was assessed before and after trimming using the fastqc online tool (https://www.bioinformatics.babraham.ac.uk/projects/fastqc/). We followed the Genome Analysis Toolkit (GATK) Best Practices recommendations^82^ to obtain variant calls from FASTQ files. Read pairs were mapped to the GRCh37 human reference genome using the Burrows-Wheeler Aligner (BWA) version 0.7.15^83^. Mapped reads were merged, and PCR duplicates were removed using the MarkDuplicates tool from Picard version 2.18 (https://broadinstitute.github.io/picard/). Coverage and mapping quality control was performed on mapped files (BAM) using GATK version 3.7^84^ before and after removing duplicates. Batch effect was assessed and discarded in this step. Indel realignment and base quality score recalibration were performed using the GATK as well (RealignerTargetCreator, IndelRealigner, BaseRecalibrator and PrintReads tools were used in this order), and SNP and indel discovery were performed with the GATK HaplotypeCaller tool. At this point, we included different worldwide populations to the dataset; 50 individuals were randomly selected from the following populations in the 1000 Genomes Project^85^ panel: Yoruba (YRI) from Western sub-Saharan Africa, Luhya (LWK) from Eastern sub-Saharan Africa, Utah residents (CEPH) with Northern and Western European ancestry (CEU), Han Chinese in Beijing (CHB), and Indian Telugu in the UK (ITU). We also included eight Mozabite individuals from^38^, to incorporate another North African and Amazigh population, and 47 Yemeni individuals from^86^, as a Middle Eastern proxy. The available Mozabite samples were originally mapped to the hg19 human reference genome, which slightly varies from GRCh37 mainly in some labels. We used a custom modified version of the hg19 reference to match the GRCh37 reference for the processing of Mozabite samples. Yemeni samples were whole-genome samples mapped to the GRCh38 human reference genome, so, after the previously described BAM filtering and processing was performed, BAM files were lifted over to the GRCh37 reference using CrossMap^87^. SNPs and indels were called with the HaplotypeCaller tool from GATK from the BAM files of all samples, including the previously published and the new sequences from the present study. The individual variant files from HaplotypeCaller for all samples were combined with the GenotypeGVCFs tool to obtain a single variant call format (VCF) file for the complete dataset. The intersection of the exome variants from the Yemeni whole genomes was automatically performed in the joint variant calling. SNPs and indels were recalibrated with the VariantRecalibrator and ApplyRecalibration GATK tools. Based on guidelines in^37^, we used VCFtools 0.1.14^88^ to exclude those variants that: (1) did not pass the VCFtools internal filters (not labelled as “PASS”), (2) were indels, (3) were located in sex chromosomes, (4) were not biallelic, (5) were monomorphic in our dataset, (6) had a depth of coverage < 5×, had a genome quality (GQ) < 20, (7) presented missingness > 5%, and (8) presented a Hardy Weinberg test value *p* < 10^–3^ in at least one of the populations. Low-quality samples were removed according to the following criteria: we required at least 30 × mean coverage (4 samples removed), 85% positions at a minimum 5 × coverage in the BAM file (no sample removed), a total genotype missingness lower than 10% (13 samples removed), and heterozygosity levels within the range of 4 standard deviations lower and higher than the corresponding population average (no samples removed). VCF quality controls were performed before and after applying these filters.

Although sampling was performed to avoid related individuals, an estimation of relatedness was performed. We used PLINK 2.0^89^ to filter out variants with minor allele frequencies lower than 0.01, and those at linkage disequilibrium calculated using sliding windows of 50 kb with a step size of 5 SNPs, and a square correlation coefficient (r^2^) threshold of 0.5. With this pruned variant set we assessed relatedness using KING^90^ and removed individuals so that no third-degree relationship or closer remained in the dataset. A quality control was performed as a final step of the data curation. The final dataset contained 319,297 SNPs with a mean coverage of 56 × and a mean missingness of 5.8%. Ti/Tv ratio was 2.8, an expected value for high quality exome variant datasets^91^. As described below, for the analyses that required determination of the ancestral allele, the size of this variant set was slightly reduced due to unknown ancestral state in some sites. The final sample count for each population after filtering was: 18 Amazigh Tunisians, 46 non-Amazigh Tunisians, 49 YRI, 48 LWK, 48 CEU, 50 CHB, 50 ITU, 31 Yemenis and 8 Mozabites.

### Population structure analyses

Principal component analysis (PCA) was performed with the SmartPCA tool from the EIGENSTRAT stratification correction method implemented in the EIGENSOFT software package version 6.0.1^92^. ADMIXTURE 1.3^93^ was applied in unsupervised mode to explore ancestry patterns. The number of ancestral clusters explored ranged from K = 2 to K = 10 with 50 independent runs for each K using a different random seed in each run. The cross-validation error was assessed for each run and mean values were calculated to determine the range with minimum error. To identify common modes among the different runs for each K and to visualize and plot the results, we used pong in greedy mode^94^. For both PCA and ADMIXTURE, data was first pruned for linkage disequilibrium with PLINK 2.0^89^ using the same parameters as described above for the relatedness estimation and resulting in a dataset of 260,952 variants. PLINK 2.0 and VCFtools 0.1.14^88^ were used to obtain intermediate files for the described analyses.

Pairwise Fst values between population pairs of a subset of populations including North Africans (Tunisians and Mozabites) and proxies for the surrounding regions (Yemenis, YRI and CEU) were computed with VCFtools 0.1.14. Comparisons were made first using all individuals in each population and then using only 8 randomly selected individuals per population, a threshold set by the 8 Mozabite samples available.

### Ancestral state allele determination

The ancestral and derived state of alleles at each site in our dataset was determined following the approach used in^37^, which consist in using the 6-EPO multi-alignment from Ensembl Compara version 59. Sites where the ancestral allele was unknown were removed from the dataset for all analysis apart from those described in the population structure analyses section above, resulting in a dataset of 306,942 SNPs for further analysis.

### Effective population size estimation

Inference of effective population sizes (N_e_) for all populations in the dataset was performed using the R-package NeON^95^, which bases the N_e_ calculation in LD patterns and was previously used in whole-exome data in^47^. We calculated the long-term effective population size, which is the harmonic mean of the N_e_ of the population sizes along the generations in the past, using each chromosome as replicate to calculate the mean (percentile 50th) and the confidence intervals (percentiles 5th and 95th), as provided by the Ne_CI function of the NeON package.

### TreeMix

TreeMix analysis was performed to calculate a drift parameter of each population. The data was first pruned for linkage disequilibrium using the previously mentioned parameters and an r^2^ threshold of 0.5, and then converted to the treemix format using PLINK 2.0 and custom scripts. Then we run the TreeMix software^96^ with no migration edges and no outgroup specification.

### Genetic diversity

Diversity indexes for all populations in the dataset were calculated based on the SFS for the synonymous sites following the same approach as in^37^. Statistics computed were pairwise nucleotide diversity (θπ), Watterson’s estimator (θw) and Tajima’s D with custom scripts based on^97^, and the nucleotide diversity for variable sites (πvar) based on^36^. Confidence intervals and statistical significance were computed by bootstrapping by site 1000 times dividing in each iteration the exome in 1000 blocks and taking 1000 random blocks allowing resampling, an approach that allows to consider the possible variance introduced by demographic processes^64,98^. Confidence intervals were set as the 0.025 and 0.975 quantiles of the bootstrap distribution. Statistical significance of the differences between different population pairs of interest was tested using t-tests.

Segregating sites and private segregating sites were directly calculated from the VCF dataset, using VCFtools 0.1.14^88^ to output the allele counts at each site in each population.

### Variant annotation

Deleteriousness of each variant was assessed using GERP RS scores, which stands for genomic evolutionary rate profile rejected substitution and is a method to predict the effect of allele substitutions based on sequence conservation^51,52^. GERP RS scores were collected from the Combined Annotation Dependent Depletion (CADD) online tool^99,100^. For SFS and other analyses performed at different levels of predicted deleteriousness, we divided variants according to GERP RS score categories using the same categorization as in^38,53^. We also used two other independent methods to assess deleteriousness of the variants: PolyPhen-2^101^ and CADD scores^99,100^. PolyPhen-2 scores were obtained from the Ensembl Variant Effect Predictor^54^, and CADD scores, from the CADD online tool. To categorize PolyPhen-2 scores we used the proposed categorization in the Ensembl Variant Effect Predictor and left the variants labelled as “unknown” out for the analysis using this categorization. For CADD scores we followed the recommendations in the CADD online site (https://cadd.gs.washington.edu/info) and the Ensembl Variant Effect Predictor. For analyses requiring genomic effect annotation (i.e. synonymous or missense variants), we used the Ensembl Variant Effect Predictor to annotate the variants.

### ROH analysis

Runs of homozygosity (ROH) were detected at individual level using PLINK 2.0^89^ and following the same approach as in^47^, which is optimized for whole-exome sequence analysis. Data was first pruned for linkage disequilibrium, which was detected using sliding windows of 50 kb with a step size of 5 SNPs, and a square correlation coefficient (r^2^ threshold of 0.8, keeping 271,652 variants. For ROH detection, we used sliding windows of 50 SNPs, and required a minimum of 50 consecutive SNPs at homozygous state without any heterozygous site in between to detect a ROH (PLINK options –homozyg-snp 50, –homozyg-window-het 0). The minimum ROH length to be annotated was set to 1 Mb. The rest of the PLINK parameters were set as default. We calculated the per-individual total number or ROHs, the total length (the sum of all ROH lengths), and the average length. To discern between the different demographic interpretations of ROHs depending on their length, we calculated the total number and total length of ROHs in three different length categories: 1–2.5 Mb, 2.5–5 Mb, and > 5 Mb. Statistical significance of the differences between different population pairs of interest was tested using t-tests.

We also calculated the ratio of missense homozygous derived genotypes to synonymous homozygous derived genotypes in the following different exomic regions: inside ROH tracts, outside ROH tracts, and inside the regions occupied by ROHs 1–2.5 Mb, 2.5–5 Mb and > 5 Mb respectively, resulting in 5 different regions. In each region, we calculated the mentioned ratio selecting only missense deleterious sites in different ranges of GERP RS scores. We used the same categorization as in^38,53^ and selected the three categories that predict SNPs to be deleterious (2 ≤ GERP < 4, 4 ≤ GERP < 6, and GERP ≥ 6). Allele counts needed to select homozygous sites were calculated for each individual using VCFtools 0.1.14^88^. Ratios were calculated per individual and statistical significance of the differences between categories within each population and GERP RS score category were assessed with t-tests.

### SFS and distribution of variants

Unfolded site frequency spectrum (SFS) was computed from the ancestral-state-annotated VCF file, using VCFtools 0.1.14^88^ to output the allele counts for each individual population, thinning population sizes to 8 randomly selected individuals (no differences were found between different iterations selecting 8 random individuals per population, nor when 18 individuals were considered after excluding the Mozabite sample). Derived allele counts per population were then grouped to have the number of sites at each derived allele count bin, the two fixed categories were removed, and the remaining were normalized over the total number of remaining sites. Resulting densities were plotted to visualize the SFS.

SFSs were performed using all sites and also using only variants in each different GERP RS score, CADD score and PolyPhen-2 score categories.

To test the statistical significance of the apparent increase in the difference between densities in the SFS between the two Tunisian populations as the GERP RS score increases, we bootstrapped 1000 SFS taking each time 18 random individuals from each Tunisian population (a threshold set by the available number of Tunisian Amazigh individuals) and allowing resampling of individuals. For each replicate, the SFS was computed. Then we calculated the difference in density for the first 10 categories of the SFS and performed t-tests to test determine the statistical significance of the density differences between sequential categories (from “neutral” to “moderate”, from “moderate” to “large” and from “large” to “extreme), i.e., to test if the density difference significantly increases with increasing GERP RS scores.

For analyses performed only in the two Tunisian populations, we used a dataset that did not contain Yemeni and Mozabite samples, allowing us to filter out all missing sites maintaining a substantial number of sites (227,747 after ancestral state determination) and increasing coverage to 63×.

In order to compare the fraction of deleterious variants in low and common frequencies between Tunisian populations we grouped all sites with at least one derived allele that had a deleterious GERP RS score (i.e. GERP ≥ 2) and divided them between low frequency sites (singletons and doubletons) and common sites (from tripletons to higher frequencies). 95% confidence intervals were calculated using the Wald method. This was repeated with PolyPhen-2 and CADD scores.

### Genetic load

We calculated the mutational load, which is the genetic load measured as the accumulation of deleterious mutations. To estimate the mutational load differences between population pairs we computed two widely used summary statistics based on individual genotypic data: the ratio of per-individual number of derived alleles (N_alleles_) and the ratio of homozygous derived genotypes (N_hom_). Ratios were calculated between different population pairs and through the four GERP RS score categories. N_alleles_ and N_hom_ were calculated directly from the VCF file using VCFtools 0.1.14^88^. Statistical significance and confidence intervals were calculated by bootstrapping 1000 times, dividing the variant dataset in 1000 blocks and taking 1000 random blocks at each iteration allowing resampling, to take into account demographic variance as mentioned above. Confidence intervals were set as the 0.025 and 0.975 quantiles of the bootstrap distribution. For this analysis, and following the approach in^37^, we required a *p* < 0.001 to declare significance. This analysis was also performed selecting, for variants with GERP ≥ 2, only those labelled as missense in the Ensembl Variant Effect Predictor^54^, and for those with GERP < 2, only those labelled as synonymous. The load analysis was replicated with PolyPhen-2 and CADD scores instead of GERP RS scores.

### GERP RS score load

As an additional approach to estimate the genetic load, we calculated the so-called GERP RS score load as proposed in^53^ and^36^, which consists in translating GERP RS scores, according to their category, into selection coefficients to be used in the original load formula from^32^. We grouped variants according to the mentioned GERP RS score categories and assigned to each deleterious category a selection coefficient: s = 4.5 × 10^–4^ for 2 ≤ GERP < 4, s = 4.5 × 10^–3^ for 4 ≤ GERP < 6, and s = 1 × 10^–2^ for GERP ≥ 6. Then, using VCFtools 0.1.14^88^, we calculated the frequency of each variant in every GERP RS score category and calculated the per-site mutational load using the formula from ^32^:

$$ {\text{Load }} = { 1 }{-}w = { 1 }{-} \, \left( {{1 } - { 2}q\left( {{1 }{-}q} \right)sh - sq^{{2}} } \right) $$

where *w* is the fitness of the genotype, *q* is the derived allele frequency, *s* is the selection coefficient, and *h* is the dominance coefficient, which was set to 0.5 when assuming an additive model of dominance, and to 0 when assuming a recessive model. To calculate the load across all sites we summed all per-site values in the corresponding model of dominance. Confidence intervals and statistical significance were calculated as described above.

### Enrichment analysis

The goal of enrichment analyses is to detect the presence of variants or genes related to specific diseases or traits at a higher frequency than what would be expected by chance. This analysis was performed only in Tunisian populations, and Fst values for each SNP between these two populations calculated with VCFtools 0.1.14^88^. Five subsets for each GERP RS score and PolyPhen categories were created selecting (i) first variants labelled as missense by the Ensembl Variant Effect Predictor^54^, (ii) then those with the corresponding GERP RS score (2 ≤ GERP < 4, 4 ≤ GERP < 6, and GERP ≥ 6) or PolyPhen (“possibly damaging” and “probably damaging”) category, and (iii) finally those SNPs with Fst values in the top 5% of the Fst value distribution for the given set. Then, a sixth subset was created by merging the variants resulting from the first two filters in the previous five datasets and selecting the variants with top 5% Fst values from those merged. This sixth dataset is referred in the tables as “All categories” and represents the top 5% Fst-value missense SNPs that are predicted to be deleterious by GERP RS score (thus, with GERP RS score ≥ 2) or PolyPhen (thus, labelled as “damaging”). As a final filtering step before proceeding with the over-representation analysis, we selected from each dataset only those variants with higher derived allele frequency in Tunisian Imazighen than in Tunisian non-Imazighen, filtering out those with higher frequency in Tunisian non-Imazighen. Because these variants are not present in these databases (with extremely few exceptions), we used their corresponding genes as an approximation.

Each gene set was used to look for enriched diseases, traits or biological pathways in four different public databases. We followed different criteria to select significantly enriched traits depending on the database. The Online Mendelian Inheritance in Man (OMIM) catalog^59^ and the Gene Ontology (GO) Resource^62,63^ were explored using the online tool WebGestalt or WEB-based GEne SeT AnaLysis Toolkit^102,103^, and uploading the complete list of genes in our dataset as reference. For these, the first criteria for a trait to be considered significant was to have an FDR < 0.05, as recommended by WebGestalt. We incorporated two extra significance criteria, in descending order of relevance, to classify the remaining results: traits with *p* < 0.05 and an enrichment ratio (the number of observed genes divided by the number of expected genes) higher than the mean ratio of the analysis for the corresponding database, and remaining traits that did not pass the two first criteria thresholds but still presented p-values < 0.05. The analysis in the ConsensusPathDB (CPDB)^61^ was performed using the over-representation analysis tool in the CPDB online site and uploading the complete list of genes in our dataset as reference and selecting the pathway option. In this case we followed the same criteria as in^47^ for significance of the enrichment, which is to require a q < 0.05, and included a second separate group of enriched traits that have q > 0.05 but *p* < 0.05. To explore the GWAS catalog^60^ we downloaded the full catalog associations and extracted a list of genes with associated traits, that was then matched to each of our gene lists counting the number of observed genes associated with each GWAS catalog trait. During the analysis we detected that some of these traits were also the ones appearing at highest frequency in the catalog. To tackle this question and as significance criteria, we conducted a bootstrap analysis for each of our gene subsets. The analysis consisted in performing 1000 iterations taking each time a number of randomly selected genes equal to the number of genes in the corresponding gene subset from (A) the complete list of genes in the GWAS catalog, and (B) the complete list of genes in our dataset. For these resampled gene sets we ran again the enrichment analysis, counting each time the number of appearances of each trait and resulting in 1000 frequency-of-appearance values for each trait. (A) and (B) gave extremely similar results, so we show here the results of (A). For each trait frequency-of-appearance distribution, we calculated the quantile 95 (Q95), which is the cut point value at the 95% of the bootstrap distribution, and only reported a trait as significance if the observed frequency of appearance was equal or higher than the Q95 of the bootstrap distribution for the given trait in the corresponding gene subset (i.e., if the value fell in the top 5% of the distribution).

For the genes appearing in the OMIM analysis, we checked the frequencies of associated variants in the general dataset for variants appearing in the OMIM and other variants of our dataset that were interesting because of their genomic or clinical consequences and their frequency differences between Tunisian populations. Allele frequencies were checked with VCFtools 0.1.14^88^. Variants in Fst peaks were also explored and those in genes related to bone diseases or conditions (of interest because of the results of the GWAS catalog analysis) were reported in Table S9 online. To define a peak, we required a SNP in the top 1% of the Fst distribution with at least one other top 1% SNP at a distance of less than 10 kb, although from the four reported peaks in Table S9 online, three of them present the top 1% SNPs at a distance of less than 1 kb from each other. Linkage disequilibrium between SNPs in these peaks was calculated using VCFtools^85^. Finally, SNPs with high frequency differences and high GERP RS score load were also selected and their corresponding genes were matched with the GWAS catalog to explore its associations.

## Supplementary Information

Supplementary Information 1.

Supplementary Information 2.

## Data Availability

Tunisian Imazighen and non-Imazighen whole-exome sequences are deposited at EGA accession number: EGAS00001005205.
