# Supplementary material for: Whole-exome analysis in Tunisian Imazighen and Arabs shows the impact of demography in functional variation
Source: Sci Rep. 2021 Oct 26;11:21125. doi: 10.1038/s41598-021-00576-0 (PMC8548440; doi:10.1038/s41598-021-00576-0)
Supplement: Supplementary file 1 — Supplementary Information 1. [file 41598_2021_576_MOESM1_ESM.pdf]

The impact of demography in functional variation: whole-exome analysis in Tunisian Imazighen and Arabs

Marcel Lucas-Sánchez[1] (ORCID: 0000-0001-6741-3959), Neus Font-Porterias[1], Francesc Calafell[1] (ORCID: 0000-0002-1083-9438), Karima Fadhlaoui-Zid[2,3], David Comas[1,*] (ORCID: 0000-0002-5075-0956)

[1] Institut de Biologia Evolutiva (CSIC-Universitat Pompeu Fabra), Departament de Ciències Experimentals i de la Salut, Universitat Pompeu Fabra, Barcelona, Spain

[2] Laboratory of Genetics, Immunology, and Human Pathologies, Faculty of Science of Tunis, University of Tunis El Manar, Tunis, Tunisia

[3] College of Science, Department of Biology, Taibah University, Al Madinah Al Monawarah, Saudi Arabia

[*] Correspondence author – e-mail: david.comas@upf.edu

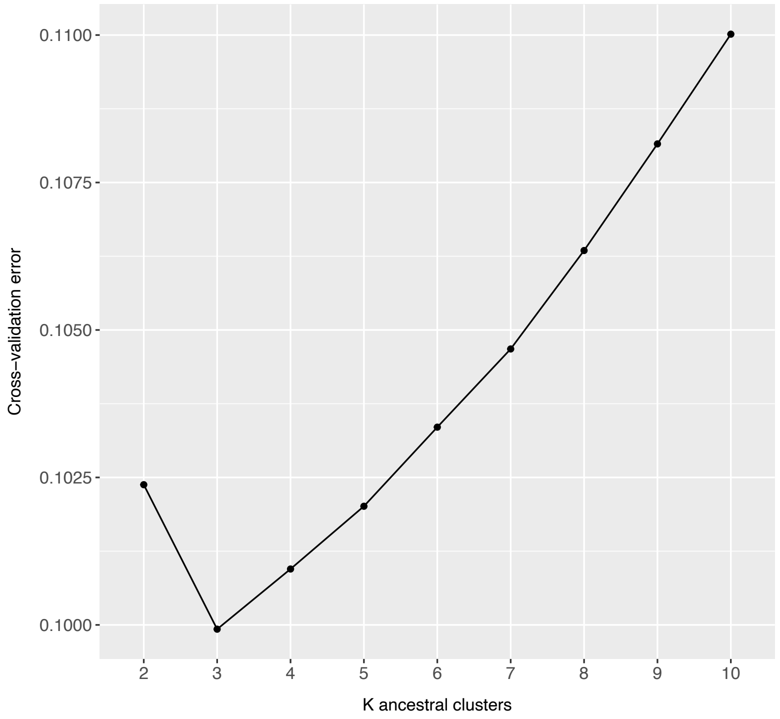

Fig. S1 ADMIXTURE Cross-validation error

Cross-validation error for the ADMIXTURE analysis in Fig. 1c.

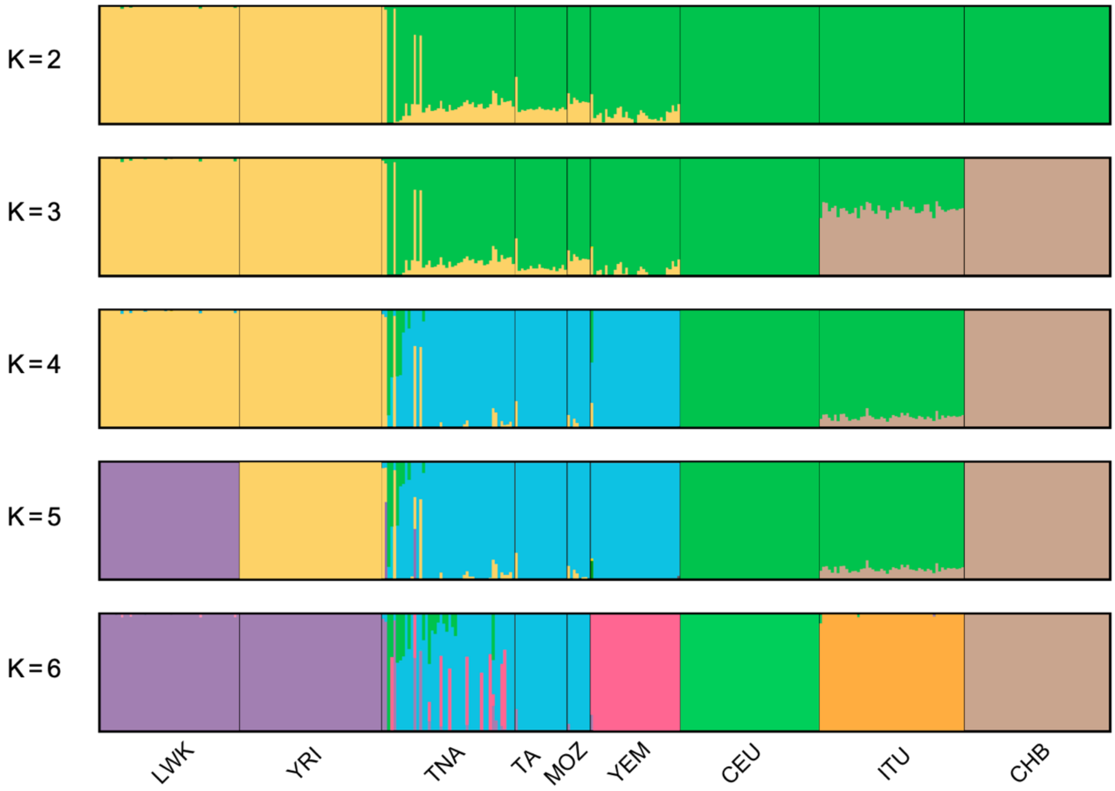

Fig. S2 ADMIXTURE analysis from K = 2 to K = 6. Related to Fig 1C

Population names abbreviated as in Fig. 1. Higher Ks show substructure in sub-Saharan populations.

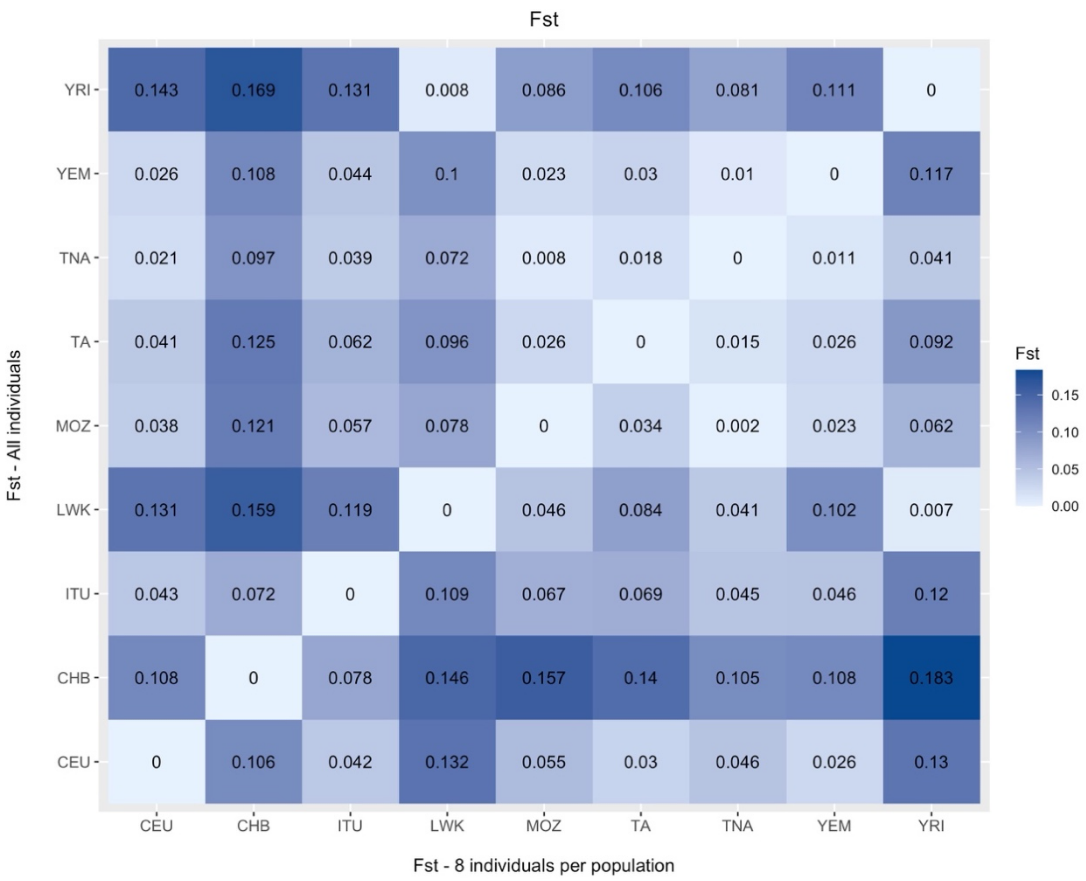

Fig. S3 Pairwise Fst values

Darker colors indicate higher Fst values. These were calculated using the program VCFtools [1]. Calculations were performed using all individuals for the chosen populations (top triangle) and also using the same number of individuals per population, 8 because of the threshold set by the 8 Mozabites (bottom triangle).

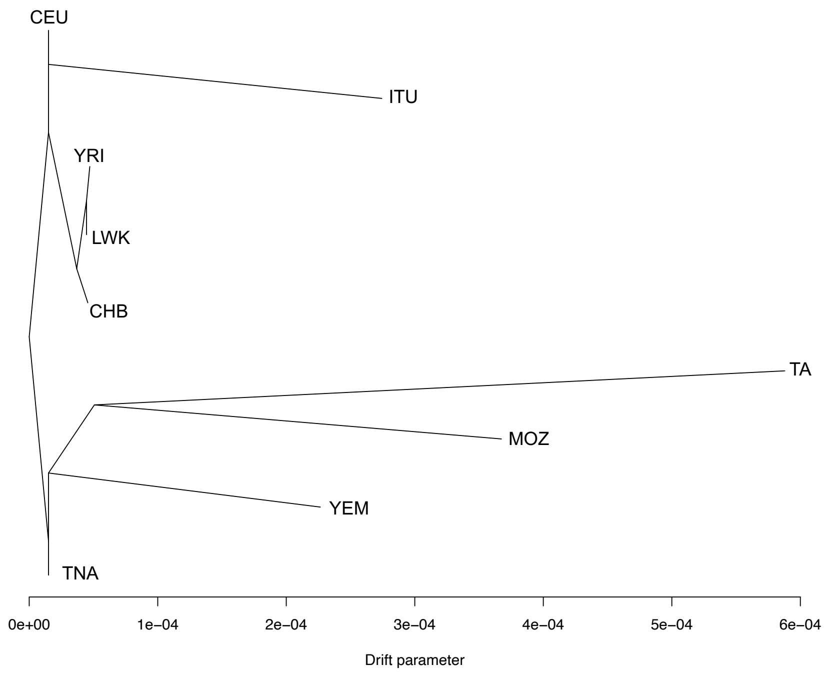

Fig. S4 TreeMix analysis of Genetic Drift
Genetic drift estimation for the different populations in the dataset calculated with TreeMix [2] accounting for 0 migration edges and with no outgroup. Population names abbreviated as in Fig. 1.

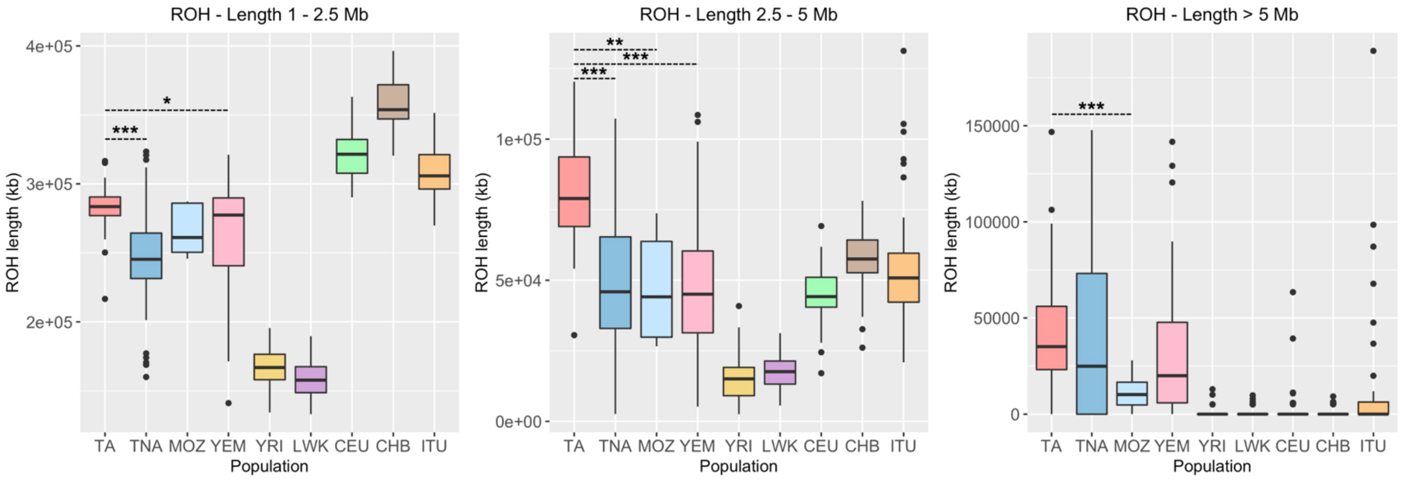

Fig. S5 Per-individual total length of runs of homozygosity in different length categories
Boxplots indicate the distribution of the per-individual total length of runs of homozygosity in different populations. Points indicate outlier individuals. Dashed lines indicate a statistically significant t-test between Tunisian Imazighen and the other North African and Middle Eastern populations. Statistical significance is shown in the following way: *p-value < 0.05, **p-value < 0.01, ***p-value < 0.001. Population names abbreviated as in Fig. 1.

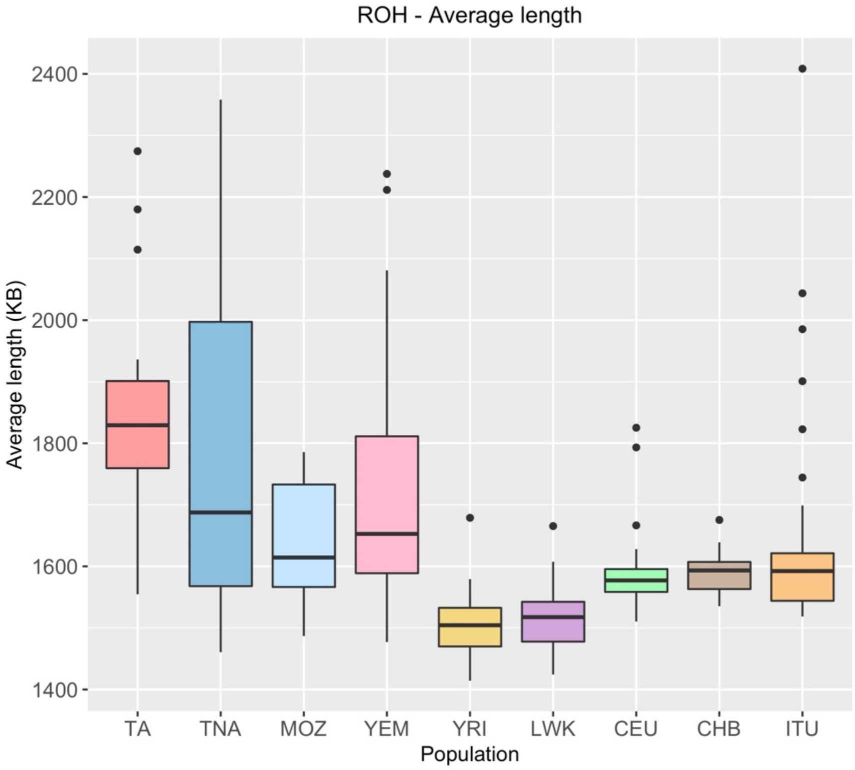

Fig. S6 Per-individual average length of runs of homozygosity
Boxplots indicate the distribution of the per-individual average length of runs of homozygosity in different populations. Points indicate outlier individuals Population names abbreviated as in Fig. 1.

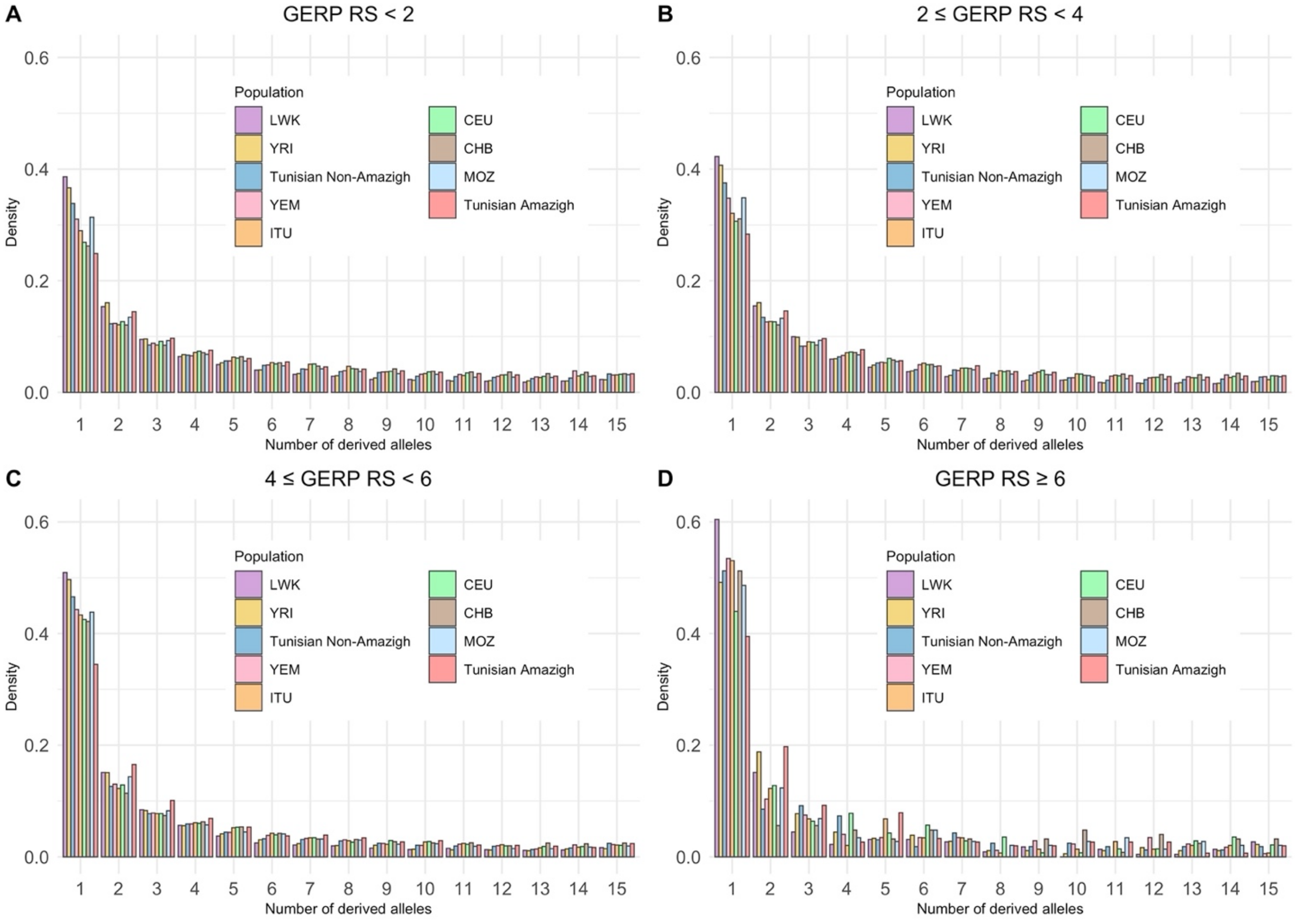

Fig. S7 Site frequency spectra of derived alleles in different GERP RS score categories

For each population, 8 randomly selected individuals were used, and fixed sites were excluded. For Mozabites, the 8 individuals selected were all the available in our dataset. Plot title indicates the range of GERP RS scores of the variants included.

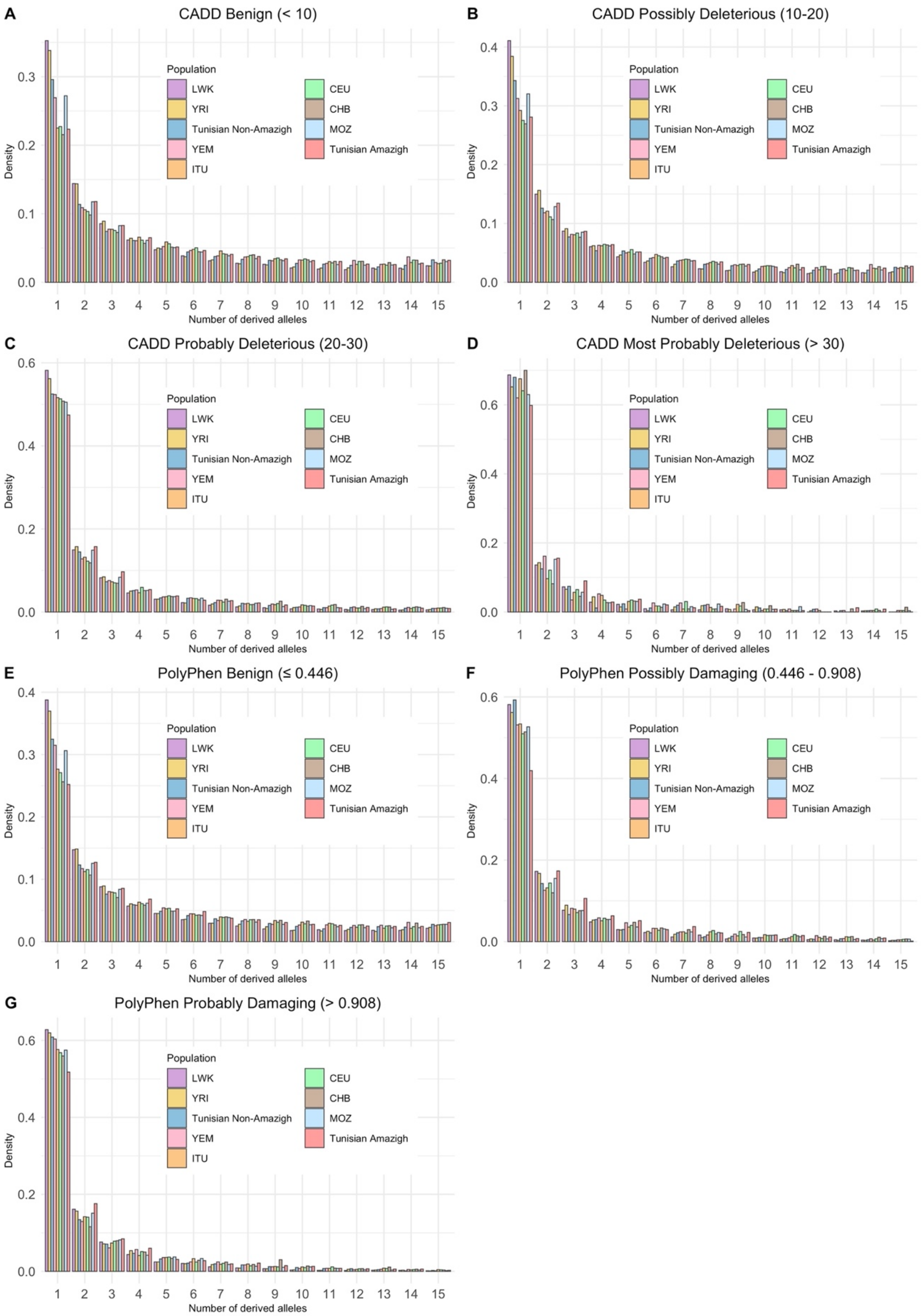

Fig. S8 Site Frequency Spectra of derived alleles in different deleteriousness categories

For each population, 8 randomly selected individuals were used, and fixed sites were excluded. For Mozabites, the 8 individuals selected were all the available in our dataset. Plot title indicates the range of (A)-(D) CADD scores and (E)-(G) PolyPhen scores of the variants included.

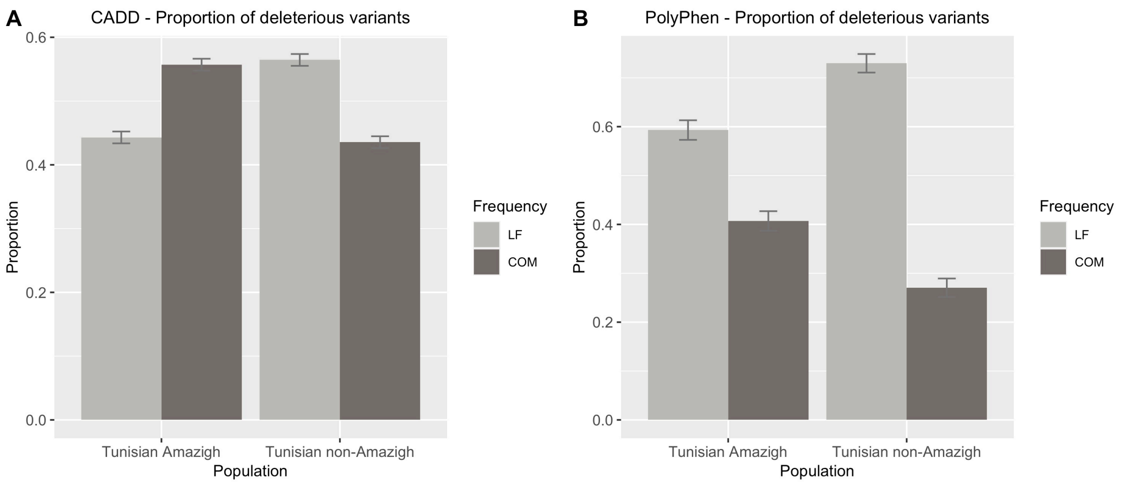

Fig. S9 Proportion of deleterious variants classified by frequency-based categories with additional methods to assess deleteriousness

(A) Includes variants with CADD scores higher than 10. (B) Includes variants labelled as Possibly Damaging or Probably Damaging in PolyPhen. The frequency-based categories are Low-Frequency (LF), including singletons and doubletons, and Common (COM), including frequencies higher than tripletons. Population included are only Tunisians. Error bars represent the 95% confidence intervals.

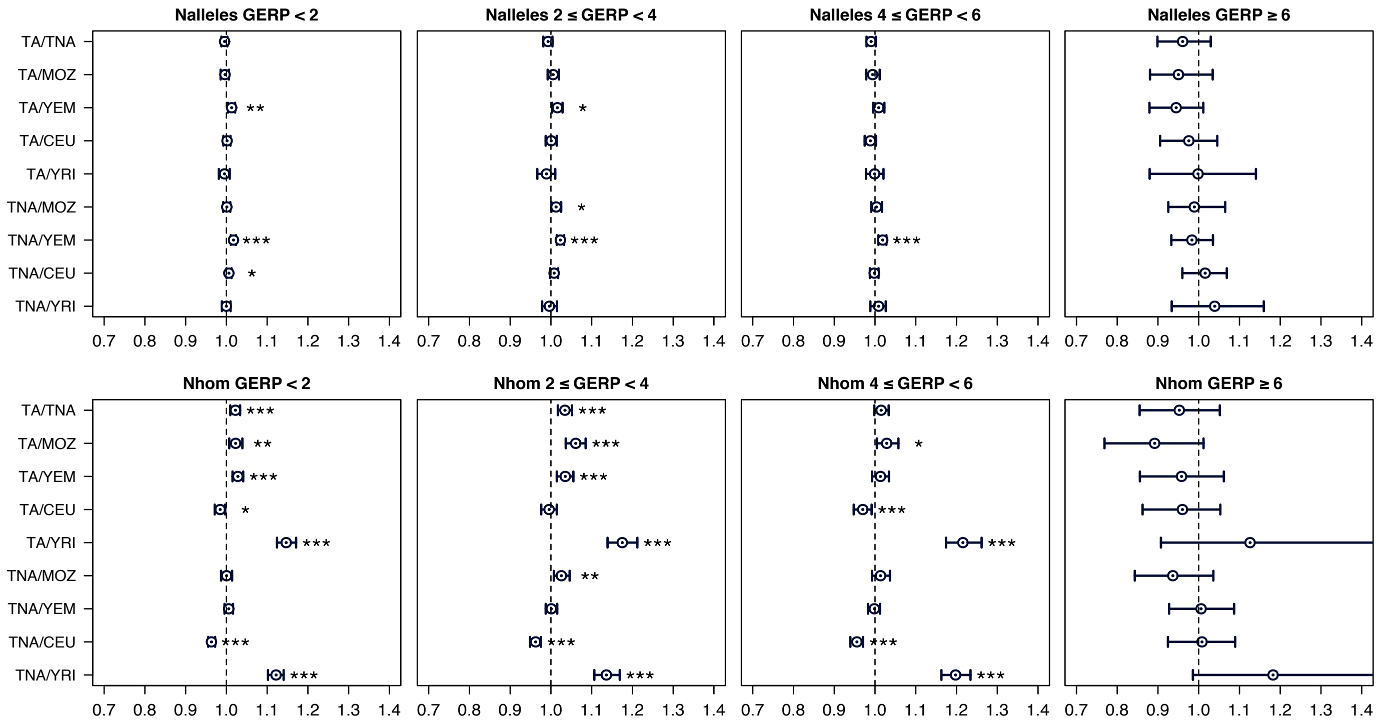

Fig. S10 Comparison of the per-individual number of derived alleles ($N_{alleles}$) and homozygous derived genotypes (N_{hom}) across populations for variants in different GERP RS score categories with the missense-synonymous filter

Pairwise population ratios of the mean per-individual number of derived alleles and homozygous derived genotypes using only GERP RS score as functional filter. Plot title indicates the range of GERP RS scores of the variants included. For GERP < 2, only synonymous variants are included. For GERP ≥ 2, only missense variants are included. Error bars represent the 0.025 and 0.975 quantiles obtained by bootstrapping by site 1,000 times, dividing the exome data into 1,000 blocks and performing bootstrap resampling of blocks 1,000 times. Statistical significance is shown in the following way: *p-value < 0.05, **p-value < 0.01, ***p-value < 0.001. To account for multiple testing errors, significance threshold was set to p < 0.001. Population names abbreviated as in Fig. 1.

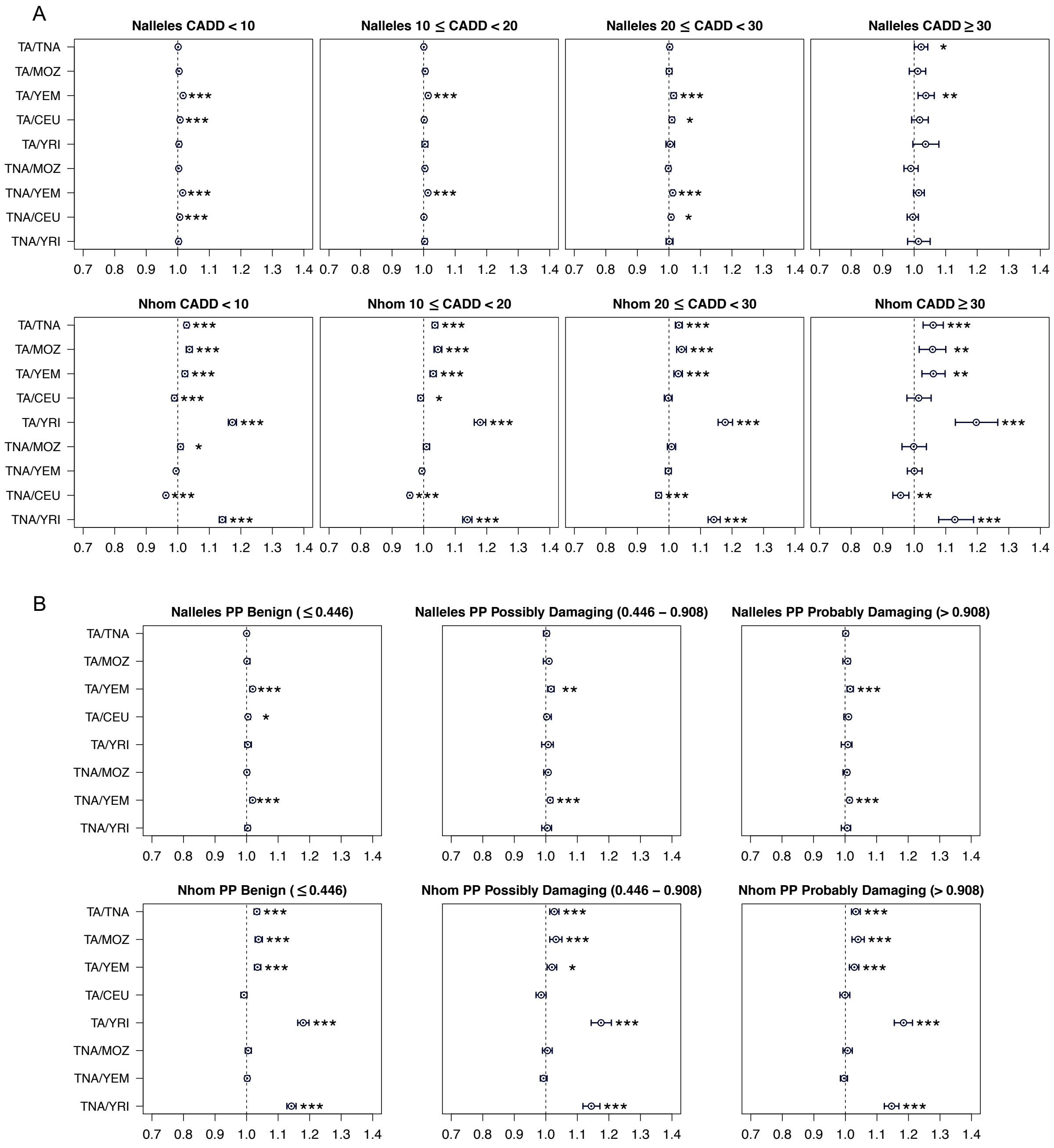

Fig. S11 Comparison of the per-individual number of derived alleles (Nalleles) and homozygous derived genotypes (Nhom) across populations for variants in different deleteriousness categories
Between-population ratios of the mean per-individual number of derived alleles and homozygous derived genotypes. Plot title indicates the range of (A) CADD scores and (B) PolyPhen scores of the variants included. Error bars represent the 0.025 and 0.975 quantiles obtained by bootstrapping by site 1,000 times, dividing the exome data into 1,000 blocks and performing bootstrap resampling of blocks 1,000 times. Statistical significance is shown in the following way: *p-value < 0.05, **p-value < 0.01, ***p-value < 0.001. Population names abbreviated as in Fig. 1.

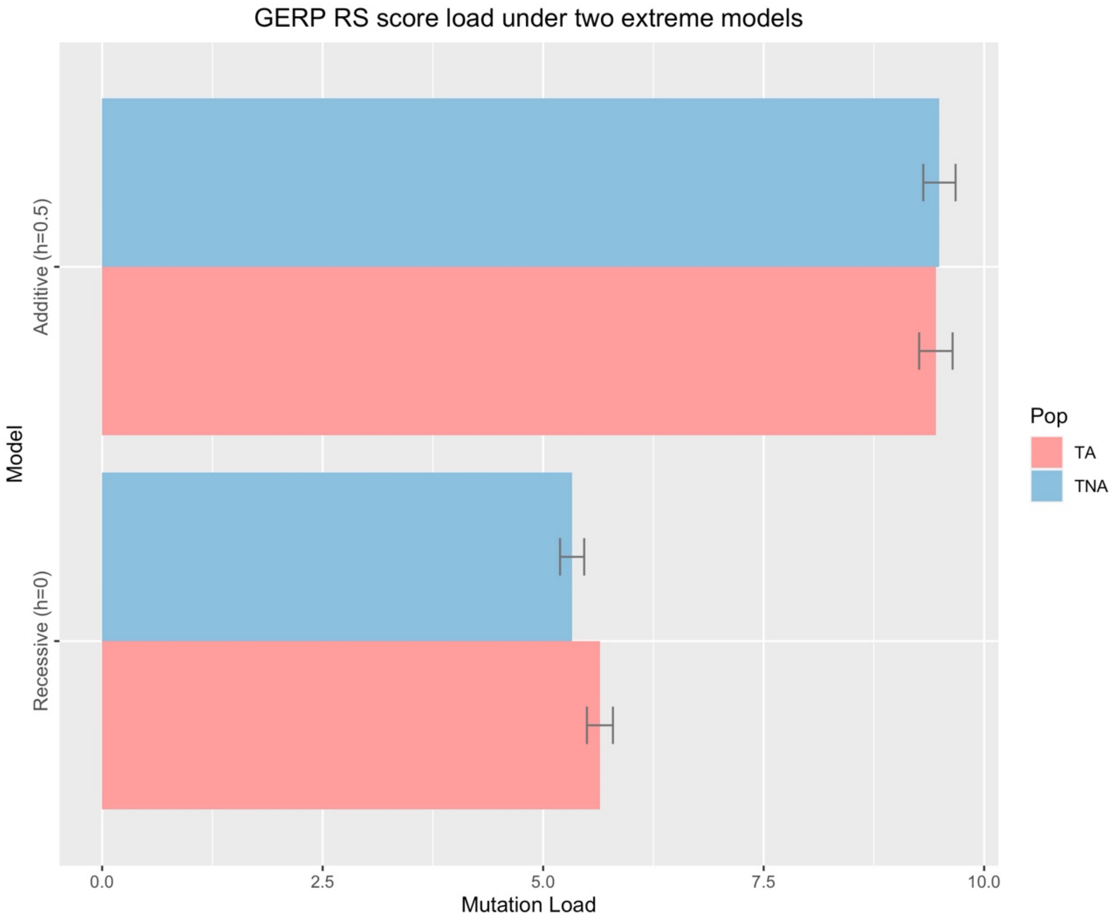

Fig. S12 GERP RS score load

GERP RS score load assuming a fully additive or a fully recessive model calculated with the load formula from Kimura et al [3] converting GERP RS scores into selection coefficients following the approach used by Henn et al [4] and Pedersen et al [5]. Error bars represent the 0.025 and 0.975 quantiles obtained by bootstrapping by site 1,000 times, dividing the exome data into 1,000 blocks and performing bootstrap resampling of blocks 1,000 times. Population names abbreviated as in Fig. 1.

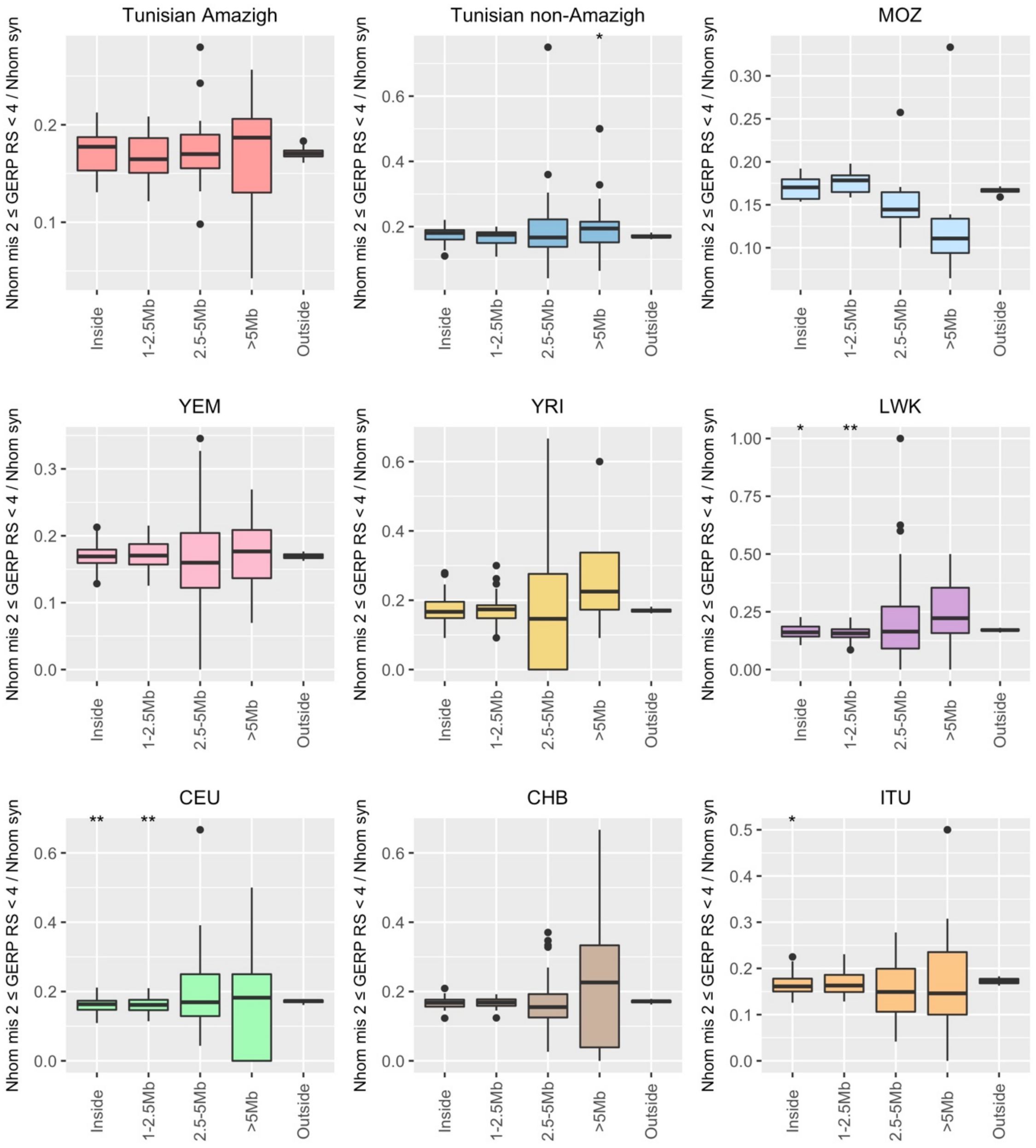

Fig. S13 Ratio of missense 2 ≤ GERP RS score < 4 to synonymous homozygous sites inside and outside ROHs

Boxplots indicate the distribution of the per-individual ratio of missense homozygous sites with 2 ≤ GERP RS scores < 4 to synonymous homozygous sites in four different regions of the exome (left to right): inside all ROH regions, inside ROHs 1-2.5 Mb long, inside ROHs 2.5-5 Mb long, inside ROHs >5 Mb long and outside ROH regions. Statistical significance is shown in the following way: *p-value < 0.05, **p-value < 0.01, ***p-value < 0.001, ****p-value < 0.0001. Population names abbreviated as in Fig. 1.

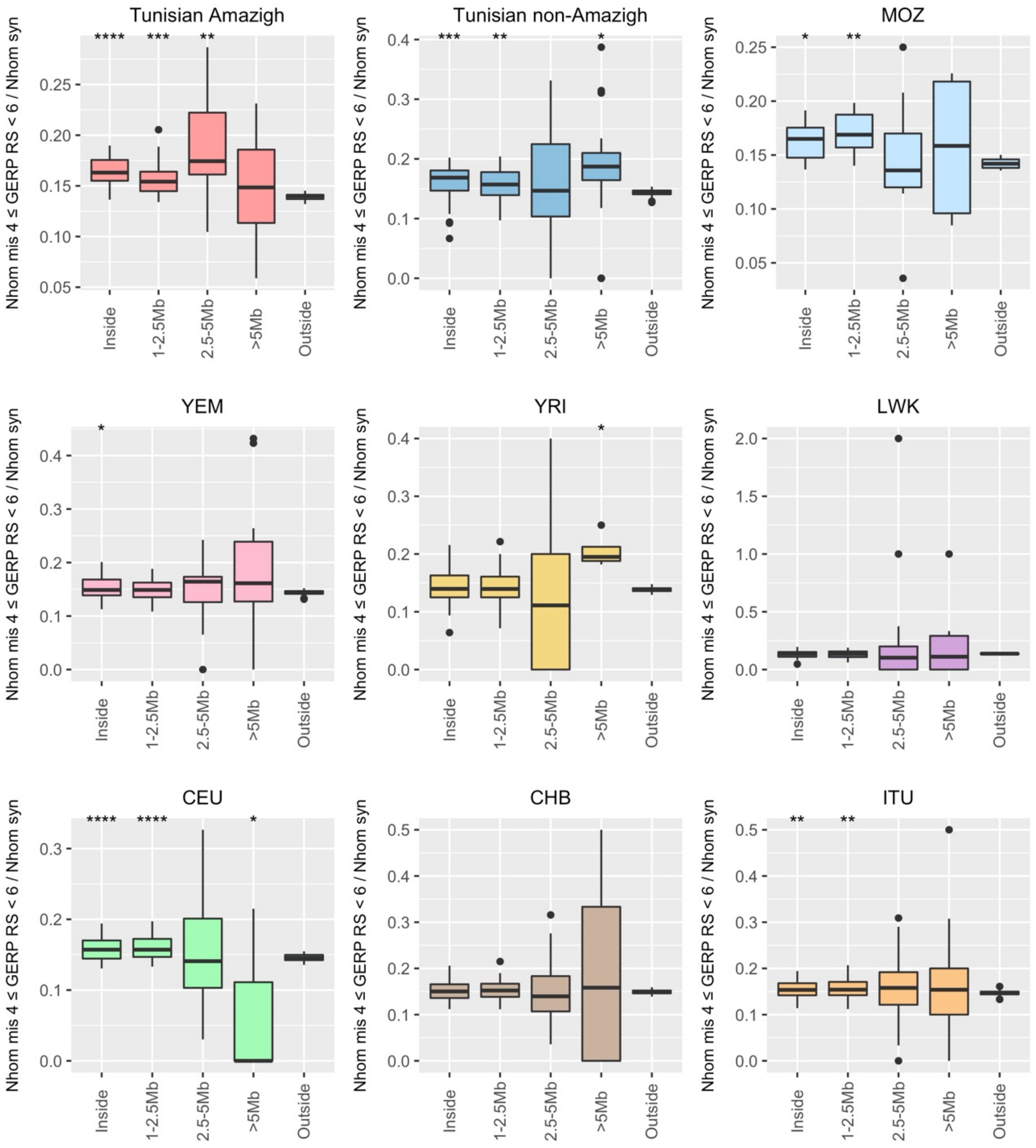

Fig. S14 Ratio of missense 4 ≤ GERP RS score < 6 to synonymous homozygous sites inside and outside ROHs

Boxplots indicate the distribution of the per-individual ratio of missense homozygous sites with 4 ≤ GERP RS scores < 6 to synonymous homozygous sites in four different regions of the exome (left to right): inside all ROH regions, inside ROHs 1-2.5 Mb long, inside ROHs 2.5-5 Mb long, inside ROHs >5 Mb long and outside ROH regions. Statistical significance is shown in the following way: *p-value < 0.05, **p-value < 0.01, ***p-value < 0.001, ****p-value < 0.0001. Population names abbreviated as in Fig. 1.

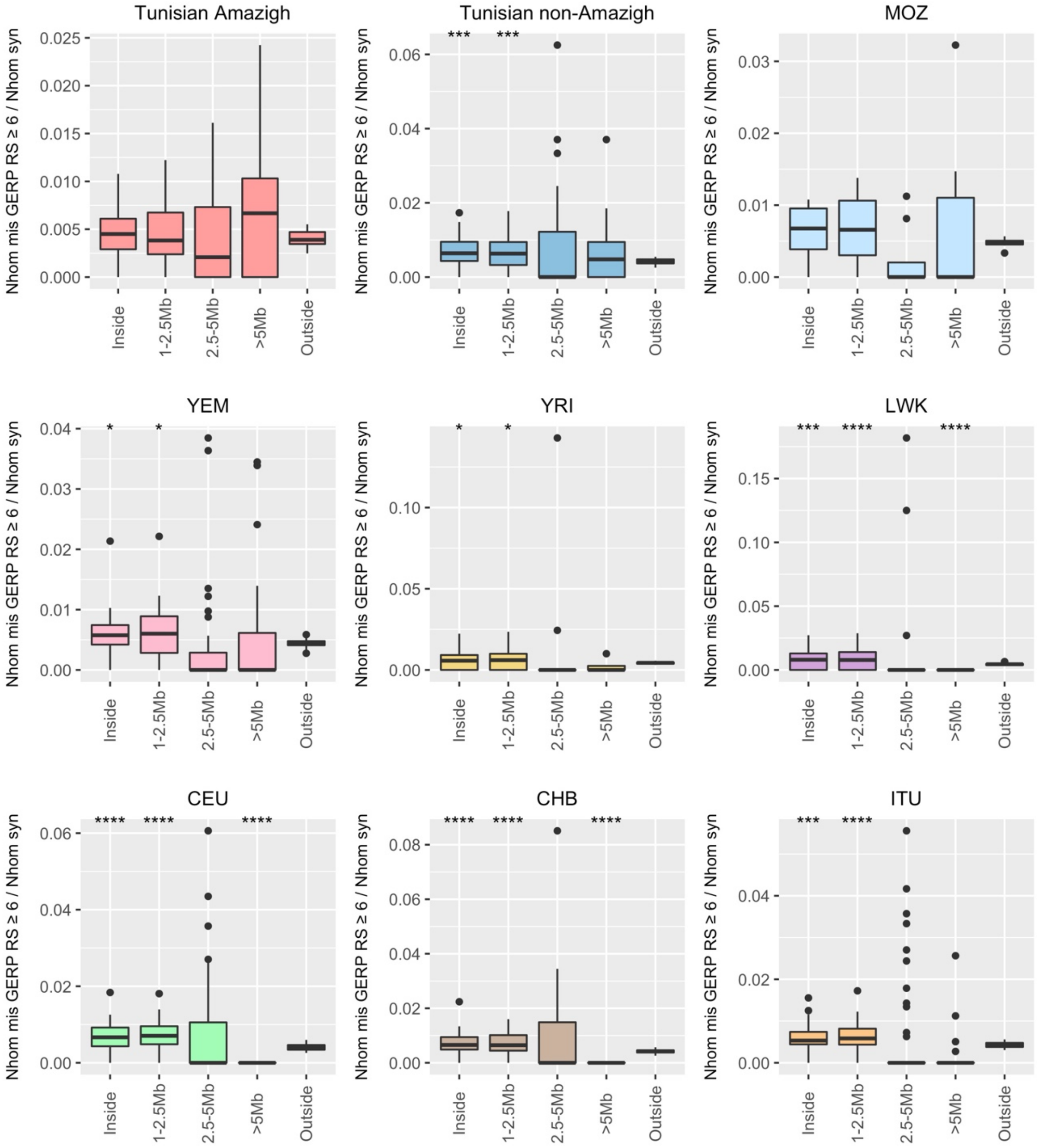

Fig. S15 Ratio of missense GERP RS score ≥ 6 to synonymous homozygous sites inside and outside ROHs

Boxplots indicate the distribution of the per-individual ratio of missense homozygous sites with GERP RS scores ≥ 6 to synonymous homozygous sites in four different regions of the exome (left to right): inside all ROH regions, inside ROHs 1-2.5 Mb long, inside ROHs 2.5-5 Mb long, inside ROHs >5 Mb long and outside ROH regions. Statistical significance is shown in the following way: *p-value < 0.05, **p-value < 0.01, ***p-value < 0.001, ****p-value < 0.0001. Population names abbreviated as in Fig. 1.

References

1. Danecek, P. *et al.* The variant call format and VCFtools. *Bioinformatics* **27**, 2156–2158 (2011).
2. Pickrell, J. K. & Pritchard, J. K. Inference of Population Splits and Mixtures from Genome-Wide Allele Frequency Data. *PLoS Genet.* **8**, 1002967 (2012).
3. Kimura, M., Maruyama, T. & Crow, J. F. The Mutation Load in Small Populations. *Genetics* **48**, 1303–1312 (1963).
4. Henn, B. M., Botigué, L. R., Bustamante, C. D., Clark, A. G. & Gravel, S. Estimating the mutation load in human genomes. *Nat. Rev. Genet.* **16**, 333–343 (2015).
5. Pedersen, C. E. T. *et al.* The effect of an extreme and prolonged population bottleneck on patterns of deleterious variation: Insights from the Greenlandic Inuit. *Genetics* **205**, 787–801 (2017).
